# Two distinct polymorphisms in the basic region of Meq protein of marek’s disease virus alter pathological progression and clinical manifestations

**DOI:** 10.1186/s12985-025-02930-4

**Published:** 2025-09-26

**Authors:** Jumpei Sato, Aoi Kurokawa, Yoshinosuke Motai, Shunsuke Yamagami, Shwe Yee Win, Fumiya Horio, Hikaru Saeki, Naoya Maekawa, Tomohiro Okagawa, Benedikt B. Kaufer, Nikolaus Osterrieder, Mark S. Parcells, Satoru Konnai, Kazuhiko Ohashi, Shiro Murata

**Affiliations:** 1 Department of Disease Control, Faculty of Veterinary Medicine, Hokkaido University, Sapporo, Japan; 2 National Institute of Animal Health, National Agriculture and Food Research Organization, Tsukuba, Japan; 3 Department of Advanced Pharmaceutics, Faculty of Veterinary Medicine, Hokkaido University, Sapporo, Japan; 4 Institut für Virologie, Freie Universität Berlin, Berlin, Germany; 5 Tierärztliche Hochschule Hannover, Hannover, Germany; 6 Department of Animal and Food Sciences, University of Delaware, Newark, USA; 7 Institute for Vaccine Research and Development, Hokkaido University, Sapporo, Japan; 8 Veterinary Research Unit, International Institute for Zoonosis Control, Hokkaido University, Sapporo, Japan; 9 International Affairs Office, Faculty of Veterinary Medicine, Hokkaido University, Sapporo, Japan

**Keywords:** Marek’s disease, Marek’s disease virus, Meq, Polymorphisms, Virulence, Oncogenicity

## Abstract

**Background:**

Marek’s disease virus (MDV) causes Marek’s disease (MD) in chickens, which is characterized by malignant lymphomas and neurological disorders. Although MD is currently controlled using live vaccines, the virulence of field strains has continuously increased in recent decades. Polymorphisms in the MDV-encoded oncoprotein Meq are shared among field strains according to their virulence. In particular, very virulent MDV strains harbor characteristic amino acid changes in the basic region of Meq at positions 77 and 80; however, the contribution of these polymorphisms to virulence remains unclear.

**Methods:**

To assess the impact of these polymorphisms on MDV virulence, we generated recombinant MDV (rMDV) based on the very virulent RB-1B strain, harboring K77E and D80Y substitutions in Meq found in low-virulent strains (rRB-1B_Meq77/80). Chickens were challenged with rMDVs, and survival rates and tumor incidence were evaluated. Viral loads in major organs were quantified by quantitative PCR, and the dynamics of MDV-infected cells and T cells were analyzed using flow cytometry. In addition, histopathological analysis was performed to further examine differences in pathogenesis in detail. To elucidate the mechanisms underlying pathogenesis, we conducted reporter assays to assess the effect of these polymorphisms in the basic region on its transcriptional regulatory activity.

**Results:**

rRB-1B_Meq77/80 exhibited a reduced virulence but unexpectedly caused other clinical signs, including open-mouth breathing, in infected chickens. Quantitative PCR analysis showed consistently lower viral loads across all examined organs in rRB-1B_Meq77/80-infected chickens. Flow cytometric analysis revealed a reduction in MDV-infected cells, accompanied by a notable increase in CD8⁺ T cell populations. Histopathological analysis showed bronchus-associated lymphoid tissue hyperplasia in the lungs. Reporter assays revealed that most amino acid substitutions in the basic region in low-virulence strains reduced transcriptional regulatory activity.

**Conclusion:**

Our data indicate that polymorphisms at positions 77 and 80 in the Meq of low-virulence strains reduce MDV virulence and Meq-mediated transcription and possibly alter pathogenesis. This study improves our understanding of the mechanisms underlying MDV virulence.

**Supplementary Information:**

The online version contains supplementary material available at 10.1186/s12985-025-02930-4.

## Background

Marek’s disease virus (MDV) is a widespread alphaherpesvirus that belongs to the Orthoherpesviridae family (subfamily: Alphaherpesvirinae; genus: *Mardivirus*; species: *Mardivirus gallidalpha 2*) (https://talk.ictvonline.org). MDV is the etiological agent of Marek’s disease (MD) and causes neurological disorders, malignant lymphomas, and immunosuppression in infected chickens. MD has caused severe economic losses to the poultry industry, but the occurrence of MD is currently reduced by vaccinating chickens with attenuated MDV and/or non-pathogenic vaccine viruses [1]. However, the virulence of field strains has been continuously increasing, resulting in sporadic MD outbreaks, even in vaccinated flocks in certain regions [2–7]. The evolution of MDV strains toward higher virulence is considered a result of adaptation to vaccine-induced immune pressure [8–11]. Currently, MDV virulence is classified into four pathotypes based on the ability to cause clinical signs in vaccinated chickens: mild (mMDV), virulent (vMDV), very virulent (vvMDV), and very virulent plus (vv + MDV) [10]. Consequently, there is ongoing concern regarding the potential for future outbreaks caused by highly virulent MDV strains [10].

MDV pathogenicity has been described in the Cornell model of MDV infection proposed by Calnek in 2001, and consists of four infection phases [12]. Airborne cell-free MDV infects chickens through the respiratory tract upon inhalation of contaminated dust and dander [13]. First, MDV infects macrophages and B cells residing in the lungs of infected chickens [14]. Macrophages transport the virus to primary and secondary lymphoid tissues, such as the thymus, bursa of Fabricius, and spleen [14–16]. MDV then semi-productively replicates in B, T, and NK cells in these primary lymphoid organs, leading to the early and transient atrophy of these organs in chickens [17–19]. Following lytic replication, MDV establishes a latent infection in CD4^+^ T cells, while a few of these cells are subsequently transformed, resulting in lethal lymphomas [20]. The rapid and highly efficient lymphoma formation is a feature of MDV pathogenesis. Most MDV-transformed tumor cells are CD4^+^ T cells, resulting from an (oligo)clonal expansion of transformed CD4^+^ T cells [20, 21].

The MDV oncogene *meq* encodes a 339-amino-acid Meq protein, which is expressed in both the lytic and latent phases of infection [22]. A *meq*-deleted recombinant MDV (rMDV) failed to cause lymphoma in infected chickens, indicating that Meq plays an essential role in the transformation induced by oncogenic MDV [23]. Meq is a basic leucine zipper (bZIP) transcription factor that regulates the expression of various viral and host genes associated with pathogenesis. Meq contains a basic region and a ZIP motif within the N-terminal domain, as well as a transactivation domain characterized by proline-rich repeats (PRRs) in the C-terminus [22]. The basic region is involved in nuclear and nucleolar localization and binding to genomic DNA [22]. The bZIP motif in Meq is similar to that of the oncoproteins Jun and Fos, allowing Meq to form homodimers with itself or heterodimers with AP-1 family proteins such as c-Jun, JunB, and Fos through ZIP [24]. Meq upregulates genes involved in the v-Jun transformation pathway, such as *JTAP-1*, *JAC*, and *HB-EGF*, in transformed DF-1 cells [25]. Meq also increases the expression of *bcl-2* and *ski* and downregulates *dap5* and *fas*, consistent with its anti-apoptotic properties [25, 26]. Furthermore, Meq interacts with various host proteins, including p53 and STING, and inhibits their function [27, 28]. Thus, Meq possesses diverse functions by binding to various proteins involved in tumorigenesis and immune evasion.

Polymorphisms in the Meq oncoprotein are shared among field strains according to their pathotypes [29]. Certain amino acid residues in the basic region and PRRs in the transactivation domain are conserved among the vvMDV and vv + MDV strains isolated in the USA. For instance, some strains classified as vvMDV in the USA, such as RB-1B and Md-5, possess an alanine at position 71, a lysine at position 77, and an aspartic acid at position 80. In contrast, low-virulence strains in the USA, such as 567 and 617 A, have a glutamic acid at position 77 and a tyrosine at position 80 [29] (Table 1). Similarly, the most recent field isolates from Asian countries, such as China and Japan, and highly virulent strains isolated in Europe and Nigeria contain glutamic acid and tyrosine at these positions in Meq, respectively [9, 11, 30–32]. Compared to the sequence of Meq in RB-1B, the Meq of the vaccine strain CVI988 contains a serine at position 71 and a glutamic acid at position 77 [29]. Furthermore, reporter assays demonstrated that amino acid substitutions in these regions significantly affect the transactivation activity of Meq [30, 33, 34]. Experimental infection using rMDVs revealed that rMDVs harboring Meq from a vvMDV or vv + MDV strain, such as RB-1B, exhibited higher virulence than those harboring Meq from a low-virulence strain, such as JM/102 W, which contains an alanine at position 77 [35]. Additionally, rMDV harboring Meq from CVI988 containing a serine at position 71 and a glutamic acid at position 77, did not induce clinical signs, such as tumor formation or neurological symptoms [36]. These observations suggest that the amino acid sequences in the basic region of Meq are closely associated with MDV virulence. However, these rMDVs also possess some polymorphisms in other domains of Meq, and this hypothesis has not been examined directly via experimental infection using rMDVs with polymorphisms only in the basic region, including those at positions 77 and 80.

**Table 1:** Comparison of the amino acid sequences of Meq in various marek’s disease virus strains

Strain	Accession No.	Virulence	Country	Position		
				71	77	80
648a	AY362725	vv+	US	A	K	D
Md5	AY243438	vv	US	A	K	D
RB-1B	AY243332	vv	US	A	K	D
CVI988	AY243333-8	m	Netherlands	S	E	D
C12/130	FJ436097	hv	UK	A	E	Y
HN302	0P897489	vv	China	A	E	Y
567	AY362709	v	US	A	E	Y
LEC-LG	OR592064	N.D.	Nigeria	A	E	Y
Kgs-c1	LC589272	N.D.	Japan	A	E	Y

Adopted from Murata et al., 2020;Yu et al., 2013; Shamblin et al., 2004. N.D.; Not determined

m, mild MDV; v, virulent MDV; vv, very virulent MDV; vv+, very virulent plus MDV, hv, hypervirulent MDV

In this study, we investigated the effects of polymorphisms at positions 77 and 80 in the basic region of Meq on MDV virulence and protein function. Specifically, we assessed the difference in the virulence between rMDV harboring RB-1B-Meq and Meq77/80 containing glutamic acid at position 77 and tyrosine at position 80 by analyzing (i) the proliferation of MDV-infected CD4^+^ T cells, the targets for MDV transformation, (ii) CD8^+^ T cells and γδ T cells, which have protective functions against MD, and (iii) histopathological findings in rMDV-infected chickens. To investigate the association between specific amino acid substitutions and the transcriptional regulatory ability of Meq, we generated expression vectors of Meq with mutations in the basic region and performed reporter assays using promoters directly targeted by Meq.

## Materials and methods

### Ethics statement

All animal experiments were approved by the Institutional Animal Care and Use Committee of Hokkaido University (approval number 22–0088). All experiments were performed in accordance with the relevant guidelines and regulations of the Faculty of Veterinary Medicine of Hokkaido University, which is fully accredited by the Association for Assessment and Accreditation of Laboratory Animal Care International.

### Cells

CEFs were obtained from 10-day-old fertilized eggs (Iwamura Hatchery Co. Ltd., Shibata, Japan) as described previously [37]. CEFs were cultured in Eagle’s minimal essential medium (Nissui Pharmaceutical Co., Ltd., Tokyo, Japan) supplemented with 10% tryptose phosphate broth (Difco Laboratories, Detroit, USA), 0.03% L-glutamine, 100 U/mL penicillin, 100 µg/mL streptomycin, 10% calf serum (Sigma-Aldrich, St. Louis, MO, USA) and 0.1% NaHCO_3_. DF-1 cells, a chicken fibroblast cell line, were cultured in Dulbecco’s modified Eagle’s medium (FUJIFILM Wako Pure Chemical Corporation, Osaka, Japan) containing 0.03% L-glutamine, 100 U/mL penicillin, 100 µg/mL streptomycin, and 10% fetal bovine serum (MP Biomedicals, Santa Ana, CA, USA) and incubated at 39 °C under 5% CO_2_.

### Generation of recombinant viruses

To investigate the effect of polymorphisms at positions 77 and 80 on virulence, we generated an RB-1B-based rMDV encoding Meq77/80, as previously described [36, 37]. To generate recombinant viruses encoding Meq or Meq77/80, we used a BAC plasmid, which had the IRL (pRB-1B_ΔIRL) [35, 36] deleted from the RB-1B genome (pRB-1B, ) [1, 2]. Subsequently, we partially deleted native *meq* in the TRL (pRB-1B_ΔIRL_ΔMeq) and inserted RB-1B-*meq* or *meq*77/80 into the *meq* locus in the TRL via two-step Red-mediated mutagenesis, as previously described [40, 41]. The resulting BAC plasmids were then subjected to RFLP analysis [37]. To screen for clones in which each *Meq* isoform was accurately inserted, the plasmids encoding each rMDV genome were digested with *Bam*HI-HF (New England Biolabs Japan Inc., Tokyo, Japan) overnight and subjected to electrophoresis on a 0.8% agarose gel, as previously described [37]. The insertion of each *Meq* isoform was further confirmed via PCR and DNA sequencing, as previously reported (40). The BAC plasmids were then transfected into CEFs using a CalPhos Mammalian Transfection Kit (Takara Bio Inc., Kyoto, Japan) according to the manufacturer’s instructions. The pCAGGS-Cre plasmid (Gene Bridges GmbH, Heidelberg, Germany) was then co-transfected to remove the BAC sequence from the viral genome. The reconstituted recombinant viruses were referred to as rRB-1B_Meq and rRB-1B_Meq77/80. All viruses were passaged on CEFs and stored in Cell Banker 1 (Nippon Zenyaku Kogyo Co., Ltd., Fukushima, Japan) at -80 °C. As the IRL region is rapidly restored during viral reconstitution (17), IRL restoration and BAC sequence deletion in each virus were confirmed via PCR (40). Each virus was titrated using plaque assays and stored as described previously [39, 42]. Finally, we sequenced the whole genomes of the reconstituted recombinant viruses using next-generation sequencing (accession number: LC862891) to confirm that they contain only the desired changes in the virus genome.

### In vitro replication of the recombinant viruses

CEFs were seeded in 12-well plates and infected with 50 pfu recombinant virus after reaching confluence. Infected cells were collected daily for 5 days. The viral loads in the infected cells were analyzed using quantitative PCR (qPCR) to assess the replication and spread of the recombinant viruses in vitro. The primer sets used are listed in Table 2.

**Table 2:** Sequences of the primers used for quantitative polymerase chain reaction

Gene	Intended use	Type	Sequence
*ICP4*	Quantification of viral loads	Forward	5´-GCATCGACAAGCACTTACGG-3´
		Reverse	5´-CGAGAGCGTCGTATTGTTTGG-3´
*iNOS*	Quantification of viral loads (endogenous control)	Forward	5´-GAGTGGTTTAAGGAGTTGGATCTGA-3´
		Reverse	5´-TTCCAGACCTCCCACCTCAA-3´
*meq*	Analysis of *meq* mRNA expression	Forward	5´-GTCCCCCCTCGATCTTTCTC-3´
		Reverse	5´-CGTCTGCTTCCTGCGTCTTC-3´
*β-actin*	Analysis of *meq* mRNA expression (endogenous control)	Forward	5´-GAGAAATTGTGCGTGACATCA-3´
		Reverse	5´-CCTGAACCTCTCATTGCCA-3´

### In vitro expression of meq using recombinant viruses

Briefly, CEFs were seeded in 12-well plates and infected with 50 pfu of recombinant viruses. Infected cells were collected daily for 5 days. The infected CEFs were then lysed with TRI reagent (Molecular Research Center, Inc., Cincinnati, OH, USA), and the total RNA was extracted according to the manufacturer’s instructions. Afterward, the total RNA was treated with DNase I (Promega), and cDNA was synthesized using PrimeScript Reverse Transcriptase (Takara Bio Inc.). The expression of each *meq* isoform in the cDNA samples was analyzed via qPCR using a TB Green Premix Dimer Eraser (Takara Bio Inc.). The primer sets used are listed in Table 2.

#### In vivo characterization of recombinant viruses

For experimental infection, fertilized eggs (Iwamura Hatchery Co., Ltd.) from commercial white leghorn chickens were hatched, and the chicks were raised in isolators at the animal facility of the Faculty of Veterinary Medicine of Hokkaido University. To analyze the recombinant viruses, the following animal experiments were performed.

### First animal experiment

The first experimental infection was performed to analyze the survival rate, tumor incidence rate, the proportion of lymphocytes in the peripheral blood mononuclear cells (PBMCs), spleen, and bursa of Fabricius, and lymphoid organ weight. A total of 103 one-day-old chicks (Iwamura Hatchery Co., Ltd.) were randomly divided into three groups (RB-1B_Meq, *n* = 36; rRB-1B_Meq77/80, *n* = 42; or phosphate-buffered saline [PBS, pH 7.4] used as a negative control; *n* = 25) and housed separately in different isolators.

#### In vivo kinetics of recombinant viruses and dynamics of T cell subsets in infected chickens

Chickens were inoculated with 5,000 pfu of rRB-1B_Meq (*n* = 20), rRB-1B_Meq77/80 (*n* = 20) via the intra-abdominal route or PBS as a negative control (*n* = 20). Five chickens per group were euthanized under deep general anesthesia via isoflurane inhalation (Zoetis Japan, Tokyo, Japan), and heparinized whole blood was collected from their hearts at 7, 14, 28, and 35 dpi. The blood, spleen, thymus, bursae of Fabricius, and feather tips were collected. DNA was extracted from the whole blood, spleen, thymus, and feather tips, and the viral loads were analyzed for each sample. Spleens, thymus, bursae, and tumor tissues were dissected with scissors, homogenized, and strained using 40-µm cell strainers (BD Biosciences, San Jose, CA, USA) to obtain cell suspensions, which were washed twice with PBS. Mononuclear cells were isolated from the whole blood, spleen, thymus, and bursa cell suspensions via density gradient centrifugation using a Percoll solution (GE Healthcare, Chicago, IL, USA), which were washed twice with PBS. The isolated cells were stored in a Cell Banker 1 (Nippon Zenyaku Kogyo Co., Ltd.) at -80℃ for flow cytometric analysis.

#### Virulence of recombinant viruses

The virulence of the recombinant viruses in infected chickens, rRB-1B_Meq (*n* = 22) and rRB-1B_Meq77/80 (*n* = 16), was compared by monitoring the disease incidence. After inoculating the chickens with these recombinant viruses, we monitored them daily for clinical signs of MD for 8 weeks. We euthanized the infected chickens showing clinical signs, such as severe weakness and feeding difficulty, during the experimental period and examined their gross tumor lesions. We also euthanized chickens exhibiting no clinical signs at 56 dpi (control group, *n* = 5; rRB-1B_Meq-infected group, *n* = 11; rRB-1B_Meq77/80-infected group, *n* = 14) and examined the tumor incidence in the infected chickens. Finally, mononuclear cells were isolated from the whole blood, spleen, thymus, and bursae of chickens euthanized at 56 dpi, as described above.

### Second animal experiment

A second experimental infection was conducted to assess the survival rate, tumor incidence, and lymphocyte proportions in the thymus and perform histopathological analyses. A total of 45 one-day-old chicks were randomly divided into three groups and housed separately. Chickens were intra-abdominally inoculated with 10,000 pfu rRB-1B_meq (*n* = 22), rRB-1B_L-Meq (*n* = 18), or PBS (*n* = 5) as a negative control. After inoculating the recombinant viruses, the clinical signs of MD were monitored daily for 56 days. We also euthanized chickens exhibiting no clinical signs at 56 dpi (control group, *n* = 5; rRB-1B_Meq-infected group, *n* = 12; rRB-1B_Meq77/80-infected group, *n* = 18) and examined tumor incidence in the infected chickens. Mononuclear cells were isolated from the whole blood, spleen, and thymus of chickens euthanized at 56 dpi, as described above. For histopathological analysis, the spleen, bursa of Fabricius, thymus, proventriculus, lungs, brain, and cervical skin were collected from each group at 56 dpi (control group: *n* = 2; rRB-1B_Meq77/80-infected group: *n* = 5; rRB-1B_Meq-infected group: *n* = 3) and fixed in 10% neutral-buffered formalin (Fujifilm Wako Pure Chemical) for 48 h.

### Quantification of viral loads using qPCR

Total cellular DNA was extracted from the whole blood, spleen, thymus, and bursae of Fabricius of infected chickens using a DNeasy Blood and Tissue Kit (Qiagen, Tokyo, Japan) according to the manufacturer’s instructions. Total cellular DNA was extracted from feather tips, as previously described [43]. Viral loads in chickens infected with recombinant viruses were determined via qPCR using primers specific for the infected cell protein 4 (*icp4*) gene of MDV. qPCR was performed using a TB Green Premix DimerEraser (Takara Bio Inc.) in a LightCycler 96 System (Roche Diagnostics, Mannheim, Germany). The chicken inducible NO synthase (*i-nos*) gene was amplified as a reference gene. Viral loads are indicated as the ratios of *icp4* to *i-nos*. The primers used for qPCR analyses are listed in Table 2. For the proliferation assay in vitro, the viral load was calculated as the mean of three independent cultures. Three independent experiments were performed.

### Flow cytometric analysis

PBMCs or mononuclear cells (5 × 10^5^) isolated from the spleen, thymus, or bursa of Fabricius were seeded into 96-well round-bottom plates and washed twice with FACS buffer (PBS supplemented with 1% bovine serum albumin; Sigma-Aldrich). The cells were then blocked with PBS containing 10% chicken serum (Thermo Fisher Scientific) at 25 °C for 15 min. Subsequently, for the gating strategy previously described in Supplementary Fig. 1, the cells were stained with mouse anti-chicken CD3ε monoclonal antibodies (mAbs) (Southern Biotech, Birmingham, AL, USA) conjugated with PerCP-Cyanine5.5 dye, mouse anti-chicken CD4 mAbs conjugated with PE-Cyanine7 dye (PE-Cy7; Southern Biotech), mouse anti-CD8α mAbs conjugated with allophycocyanin (APC; Southern Biotech), mouse anti-chicken CD8β mAbs conjugated with fluorescein-5-isothiocyanate (FITC; Southern Biotech), and mouse anti-chicken TCRγδ mAbs conjugated with phycoerythrin (PE; Southern Biotech) for 30 min at 4 °C in the dark. Dead cells were stained using a Fixable Viability Dye eFluor780 (Thermo Fisher Scientific). After washing the cells twice with PBS containing 1% bovine serum albumin (BSA), they were fixed and permeabilized by resuspending them in 200 µl of a Cytofix/Cytoperm solution (BD Biosciences) for 20 min at 4 °C for the intracellular staining of Meq [44]. After three washes with Perm/Wash buffer (BD Biosciences), the cells were stained with mouse anti-Meq mAbs [45] conjugated with APC or mouse IgG_1_ isotype mAbs (APC; Southern Biotech) for 40 min at 4 °C in the dark. After two final washes with Perm/Wash buffer, cells were resuspended in 400 µL of PBS containing 1% BSA and analyzed using a FACSLyric flow cytometer (BD Biosciences). The absolute number of cells in each T cell subset was determined via flow cytometry using counting beads (CountBright absolute counting beads; Invitrogen, MA, USA) according to the manufacturer’s recommendations and the following formula:

(number of T cell subset events/number of bead events) × number of beads added.

### Histopathological analysis of rMDV-infected chickens

Histopathological analysis was conducted following a previously described IHC protocol [45]. For IHC analysis, we used monoclonal antibodies against Meq [45] and pp38, which were generated recently in the same manner. Briefly, recombinant pp38 was expressed in *Spodoptera frugiperda* 9 (Sf9) cells using a Bac-to-Bac baculovirus recombinant protein expression system (Thermo Fisher Scientific). The *pp38* gene from the Md5 strain was amplified and cloned into the pFastbac1 vector. Recombinant baculovirus was generated by transfecting Sf9 cells with recombinant baculovirus DNA. Recombinant pp38 was prepared from baculovirus-infected Sf21 cells and used for immunization. Two 7-week-old BALB/c mice (Japan SLC, Shizuoka, Japan) were intraperitoneally injected with recombinant pp38 antigen (10 µg per dose) mixed with an aluminum hydroxide-based adjuvant (2 mg in 100 µL per dose) three times at 2-week intervals. The spleen cells of the immunized mice were fused with mouse myeloma P3U1 cells using dimethyl sulfoxide Hybri-Max reagent (Sigma-Aldrich). Hybridomas secreting anti-recombinant pp38 IgG mAbs were screened via indirect ELISA and cloned twice using single-cell sorting, as previously described [45].

Tissues from the experimentally infected chickens were fixed in 10% neutral-buffered formalin (Fujifilm Wako Pure Chemical) for 48 h and subsequently embedded in paraffin. Deparaffinized sections were incubated with 0.3% H_2_O_2_ in methanol for 20 min at room temperature (20–26 ℃) to block endogenous peroxidase activity. To reduce nonspecific antibody binding, the sections were treated with a 5% skim milk solution (Fujifilm Wako Pure Chemical) for 20 min at room temperature. Supernatants from the cloned hybridoma cell cultures containing anti-Meq mAbs [45] or anti-pp38 mAbs were used as primary antibodies. The sections were then incubated with each primary antibody (1.4 mg/ml for Meq, 4 µg/ml for pp38) for 20–24 h at 4 °C. Following PBS rinsing, the sections were treated with a horseradish peroxidase polymer-based secondary antibody reagent (Histofine Simple Stain MAX PO [M] kit, Nichirei Bioscience) for 45 min at room temperature (20–26 ℃). Antigen-antibody reactions were visualized using a DAB substrate kit (Nichirei Bioscience). The slides were counterstained with Mayer’s hematoxylin (Muto Pure Chemicals) and coverslipped. We also performed IHC analysis using the hybridoma culture supernatants without anti-Meq antibodies to rule out the possibility of nonspecific reactions due to components in the hybridoma culture supernatant. Furthermore, we conducted IHC without primary antibodies to exclude the possibility of nonspecific reactions with the secondary antibodies. In both negative controls, no signal was confirmed in the tumor tissue from rRB-1B_Meq77/80-infected chickens. Finally, the immunostaining results were observed under a microscope.

### Plasmids

Expression plasmids for each Meq isoform with mutations were constructed, and mutations were introduced via site-directed mutagenesis, as reported previously [37]. The open reading frame of *Meq* derived from RB-1B (accession number: HM488349.1) was amplified and cloned into the pCI-neo vector (Promega, Madison, WI, USA). Next, we introduced alanine-to-serine, lysine-to-glutamic acid, and aspartic acid-to-tyrosine substitutions at positions 71, 77, and 80 in *Meq*, respectively, using the primers listed in Table 3. To measure transactivation activity, we constructed a c-Jun expression plasmid [38] and reporter plasmids by inserting the *pp38* promoter, *meq* promoter, and chicken *bcl-2* promoter regions upstream of the firefly luciferase-coding region in the pGL3-Basic vector (Promega) (38). The pRL-TK *Renilla* luciferase plasmid (Promega) was used as the control plasmid.

**Table 3:** Primers used to introduce mutations into the Meq gene

Position in Meq	Substitution	Type	Sequence
71	Serine-to-alanine	Forward	5´-GAATCGTGACGCCGCTCGGAGAAGACG-3´
		Reverse	5´-CGTCTTCTCCGAGCGGCGTCACGATTC-3´
77	Glutamic-acid-to-lysine	Forward	5´-AGAAGACGCAGGGAGCAGACGTACT-3´
		Reverse	5´-AGTACGTCTGCTCCCTGCGTCTTCT-3´
80	Aspartic acid-to-tyrosine	Forward	5´-AGGAAGCAGACGTACTATGTAGACA-3´
		Reverse	5´-TGTCTACATAGTACGTCTGCTTCCT-3´

### Dual-luciferase reporter assay

First, DF-1 cells were seeded in 24-well plates at a density of 2.0 × 10^5^ cells per well and incubated for 24 h. For reporter assays using the *meq* and chicken *bcl-2* promoters, the cells in each well were transfected with 300 ng of the expression plasmids for each Meq isoform, 200 ng of the c-Jun expression plasmid, 500 ng of the reporter plasmid, and 5 ng of control pRL-TK using Lipofectamine 2000 (Thermo Fisher Scientific, Waltham, MA, USA) according to the manufacturer’s instructions. For the reporter assay using the *pp38* promoter, cells in each well were transfected with 300 ng of the expression plasmid for each Meq isoform, 500 ng of the reporter plasmid, and 5 ng of control pRL-TK using Lipofectamine 2000 (Thermo Fisher Scientific). Transfected cells were lysed 24 h after transfection using 1× Passive Lysis Buffer (Promega). Luciferase activity was measured using a Dual-Luciferase Reporter Assay System (Promega) and a Luminescencer AB-2350 Phelios (Atto Corp., Tokyo, Japan). The luminescence intensity of firefly luciferase was normalized to that of *Renilla* luciferase, and the results are reported relative to the value of luciferase activity in cells transfected with the pCI-neo vector. Three independent experiments were performed in triplicate.

### Statistical analyses

Statistical analyses were performed using R Statistical Software (version 4.0.3; R Foundation for Statistical Computing, Vienna, Austria). Multistep growth kinetics were analyzed using the Mann–Whitney U test. The dynamics of the lymphoid organ-to-body weight ratios and T cell subsets in chickens were analyzed using Dunn’s test. Tumor incidence was analyzed using Fisher’s exact test. Transactivation activity was analyzed using Tukey’s multiple comparison test. Statistical significance was set at *p* < 0.05.

## Results

### Generation and characterization of recombinant viruses in vitro

**Fig. 1:**
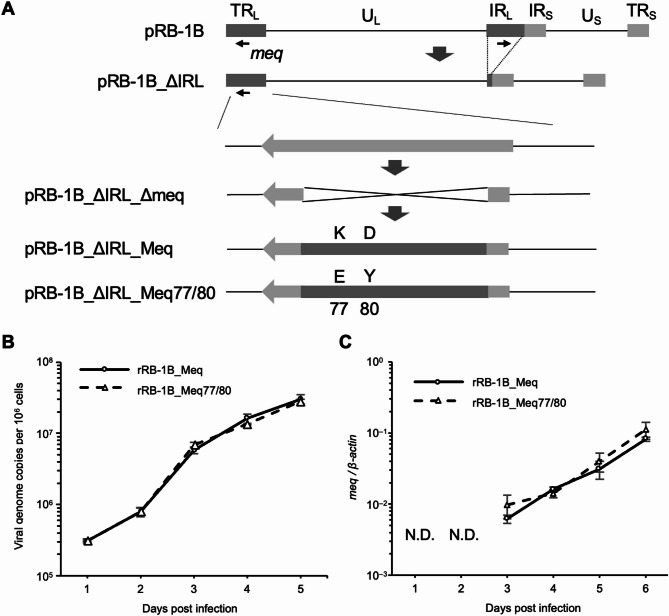
Reconstitution of recombinant Marek’s disease viruses (rMDVs) and their characterization *in vitro*. (**A**) Schematic diagrams of the constructs cloned using the RB-1B genome in this study. Most of the internal repeat long (IRL) regions were deleted in the RB-1B genome cloned as a bacterial artificial chromosome (BAC) plasmid (pRB-1B). It was then designated as pRB-1B_ΔIRL and used for mutagenesis. *Meq* in the terminal repeat long (TRL) was replaced with RB-1B-*meq* or *meq77/80* via two-step Red-mediated mutagenesis. (**B**) Chicken embryo fibroblasts (CEFs) were infected with 50 pfu of rMDVs. The infected cells were collected daily for 6 days, and the viral loads were analyzed using quantitative PCR (qPCR). (**C**) mRNA expression of each *meq* isoform in CEFs infected with rRB-1B_Meq and rRB-1B_Meq77/80 as confirmed using reverse transcription-qPCR. Three independent experiments were performed in triplicate. The growth and gene expression kinetics among the groups were analyzed using the Mann–Whitney U test. N.D., not determined

To investigate whether polymorphisms at positions 77 and 80 affect MDV virulence, we generated an RB-1B-based rMDV encoding Meq77/80, whose amino acid residues at positions 77 and 80 were substituted with those commonly found in low-virulence strains in USA and Asian isolates, as previously reported [37]. We partially deleted native *meq* in the terminal repeat long (TRL) region from a bacterial artificial chromosome (BAC) plasmid, cloned the entire genome of RB-1B (pRB-1B [39]), harboring a deletion in most of the internal repeat long (IRL) region (pRB-1B_ΔIRL [35, 36]), , which was rapidly restored upon virus reconstitution, in a BAC-based genetic system (pRB-1B_ΔIRL_Δ*meq* [46]), . Subsequently, *meq*77/80 was inserted into the *meq* locus in the TRL (pRB-1B_ΔIRL_Meq77/80, Fig. 1A). The resulting BAC plasmids were confirmed via restriction fragment length polymorphism (RFLP) analysis, PCR, and Sanger sequencing of the *meq* locus (data not shown). Subsequently, recombinant viruses (rRB-1B_Meq and rRB-1B_Meq77/80) were reconstituted in chicken embryo fibroblasts (CEFs), and IRL restoration was confirmed via PCR (data not shown). We then sequenced the whole genomes of the reconstituted recombinant viruses using next-generation sequencing (accession number: LC862891) and confirmed that no mutations were found in the ORFs of genes previously reported to be associated with MDV virulence, including *pp38*, *pp14*, and *viral interleukin-8*.

To confirm that the recombination process did not impair viral replication and *meq* expression in vitro, we analyzed the viral loads and expression of *meq* in CEFs infected with rRB-1B_Meq and rRB-1B_Meq77/80. No significant differences in the growth kinetics of the rMDVs were observed in vitro (Fig. 1B). Additionally, RT-qPCR analysis confirmed no significant differences in the transcriptional level of *meq* mRNA in vitro (Fig. 1C). These results suggest that amino acid substitutions at positions 77 and 80 do not affect viral growth kinetics or *meq* expression in cell culture.

### Virulence of rMDVs in vivo

**Fig. 2:**
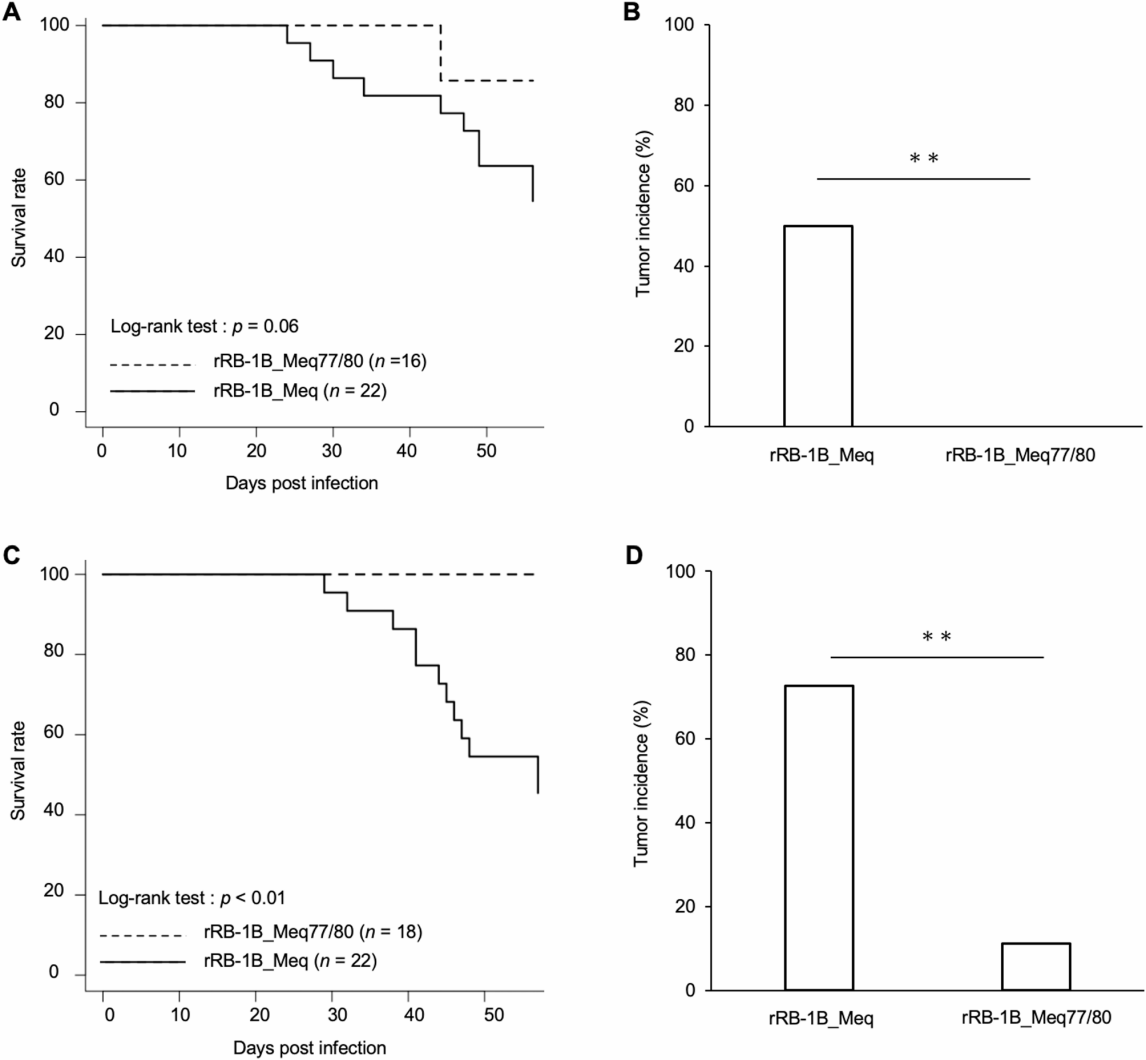
Mortality and tumor incidence in chickens infected with rMDVs. (**A, C**) Survival rate in chickens infected with rMDVs in (**A**) the first and (**C**) the second animal experiments. The survival rates among the groups were analyzed using the log-rank test. (**B, D**) Tumor incidence in chickens infected with rMDVs throughout the experimental infection. Asterisks indicate significant differences (** *p* < 0.01; Fisher’s exact test)

To assess the effect of amino acid substitutions at positions 77 and 80 on MDV virulence and tumor formation, one-day-old chickens were infected with 5,000 pfu of rMDVs via the intra-abdominal route. In the first experiment, at 56 days post-infection (dpi), 50% of the chickens infected with rRB-1B_Meq exhibited clinical signs, including leg paralysis and torticollis, and half of the chickens, including chickens euthanized at 56 dpi, exhibited solid tumor formation in the visceral organs (Fig. 2A and B). In contrast, the mortality rate in the rRB-1B_Meq77/80 group was significantly lower than that in the rRB-1B_Meq group. However, two chickens infected with rRB-1B_Meq77/80 displayed clinical signs, such as open-mouth breathing and depression. No solid tumors were detected in any chickens in the rRB-1B_Meq77/80-infected group.

To further assess the pathogenesis of rRB-1B_Meq77/80, 1-day-old chickens were challenged with 10,000 pfu of rMDVs. No dead birds were observed in the rRB-1B_Meq77/80-infected group (Fig. 2C). Although two chickens infected with rRB-1B_Meq77/80 temporarily exhibited open-mouth breathing, their manifestations were mild without wasting and feeding difficulties, and they recovered by the end of the experimental period. Moreover, solid tumors were observed in two other chickens euthanized at 56 dpi in the rRB-1B_Meq77/80-infected group (Fig. 2D). Solid tumors were observed in the spleen and liver in one chicken and in the lung, kidney, and glandular stomach of the other chicken. These data suggest that amino acid substitutions at positions 77 and 80 drastically affect virulence and alter clinical signs.

### Changes in lymphoid organ weight in chickens infected with rMDVs

The lytic replication of MDV causes a high level of cell apoptosis in the thymus and bursa of Fabricius, leading to atrophy of these organs in chickens [18]. In addition, MDV transforms CD4^+^ T cells into lymphoma cells, resulting in their proliferation and subsequent enlargement in the spleens of infected chickens [47]. To investigate the impact of rMDVs on the lymphoid organs, we measured the weight of the thymus, bursae, and spleens at each time point and calculated the weight of these lymphoid organs relative to their body weight.

**Fig. 3:**
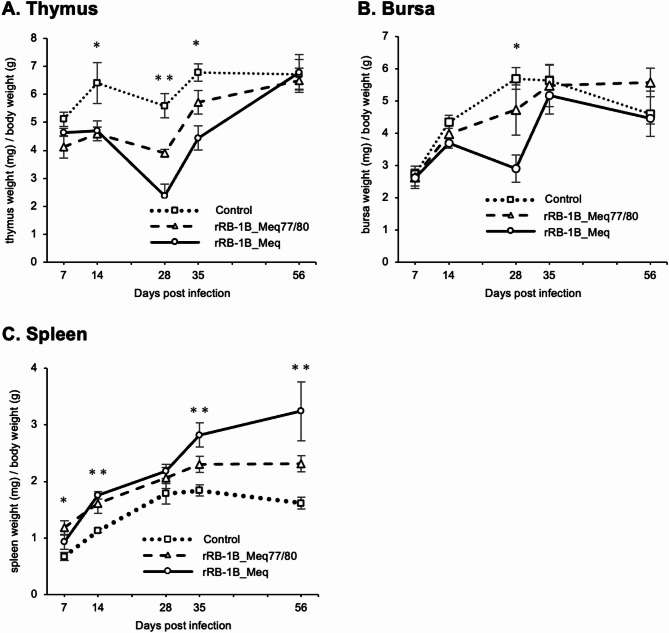
Change in lymphoid organ weight in chickens infected with rMDVs. The dynamics of the lymphoid organ-to-body weight ratios for the (**A**) thymus, (**B**) bursa of Fabricius, and (**C**) spleen in chickens infected with rRB-1B-Meq or rRB-1B-Meq77/80 were analyzed at each time point throughout the experimental period. The spleens, thymii, and bursae were collected from five chickens per group at 7, 14, 28, and 35 dpi, and from all remaining chickens (uninfected control group: *n* = 5, rRB-1B_Meq-infected group: *n* = 11, rRB-1B_Meq77/80-infected group: *n* = 14) at the termination of the experiment at 56 dpi in the first animal experiment, and 35 chickens (control group: *n* = 5, rRB-1B_Meq-infected group: *n* = 12, rRB-1B_Meq77/80-infected group: *n* = 18) at 56 dpi in the second animal experiment. Statistical significance was determined using Dunn’s test. ** *p* < 0.01 and * *p* < 0.05. Significant differences were observed at 14 dpi (rRB-1B_Meq77/80 vs. control, *p* = 0.043; rRB-1B_Meq vs. control, *p* = 0.036), 28 dpi (rRB-1B_Meq vs. control, *p* = 0.0008) and 35dpi (rRB-1B_Meq vs. control, *p* = 0.0045) in (**A**), at 28 dpi (rRB-1B_Meq vs. control, *p* = 0.0164) in (**B**), and at 7 dpi (rRB-1B_Meq77/80 vs. control, *p* = 0.0087), 14 dpi (rRB-1B_Meq77/80 vs. control, *p* = 0.029; rRB-1B_Meq vs. control, *p* = 0.0045), 35 dpi (rRB-1B_Meq vs. control, *p* = 0.0028), and 56 dpi (rRB-1B_Meq vs. control, *p* = 0.015; rRB-1B_Meq77/80 vs. rRB-1B_Meq, *p* = 0.042) in (**C**)

The weight of the thymus of chickens in both rMDV-infected groups was significantly lower than that of the control group at 14, 28, and 35 dpi (Fig. 3A), showing thymus atrophy in both infected groups. In contrast, the thymic atrophy in chickens infected with rRB-1B_Meq77/80 was milder than that in the rRB-1B_Meq-infected group at 14, 28, and 35 dpi, although the differences were not statistically significant. In addition, the bursa weight of both rMDV-infected groups was lower than that of the control group at 28 dpi (Fig. 3B). However, the bursa weight of rRB-1B_Meq77/80 was higher than that of rRB-1B_Meq-infected chickens. Thus, rRB-1B_Meq77/80 causes atrophy in the thymus and bursa, whose severity appears to be lower than that of rRB-1B_Meq. These findings suggest that two point mutations in the basic region of Meq may alter the severity of thymus and bursa atrophy.

Over time, the spleens of chickens infected with rMDV were significantly larger than those in the control group (Fig. 3C). However, the spleens of chickens infected with rRB-1B_Meq77/80 tended to weigh less at 35 dpi and significantly less than that of the rRB-1B_Meq-infected group at 56 dpi ( rRB-1B_Meq77/80 vs. rRB-1B_Meq, *p* = 0.042). Thus, the severity of the pathogenesis in the spleens of rRB-1B_Meq77/80-infected chickens was decreased compared to that of rRB-1B_Meq-infected chickens.

In vivo **replication of rMDVs**.

**Fig. 4:**
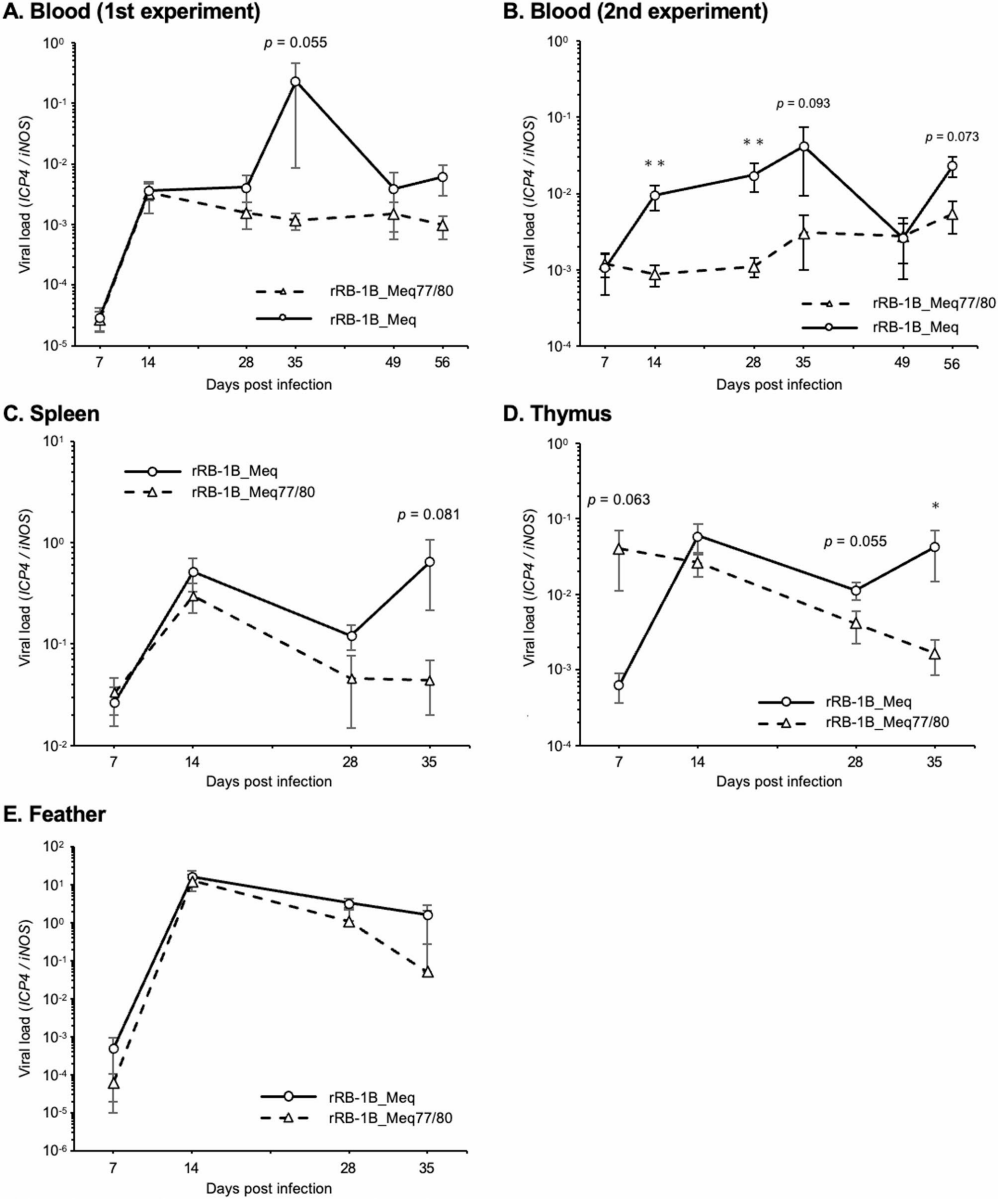
Replication of recombinant MDVs in vivo. Viral loads in (**A**) whole blood in the first experiment, (**B**) whole blood in the second experiment, and in the (**C**) spleen, (**D**) thymus, and (**E**) feather tips from chickens infected with rRB-1B_Meq or rRB1B_Meq77/80 in the first experiment were determined using qPCR. Blood samples were collected from five chickens per group at 7, 14, 28, 35, and 49 dpi, and from 25 chickens in the first experiment (rRB-1B_Meq-infected group: *n* = 11, rRB-1B_Meq77/80-infected group: *n* = 14) and 30 chickens in the second experiment (rRB-1B_Meq-infected group: *n* = 12, rRB-1B_Meq77/80-infected group: *n* = 18) at 56 dpi. Asterisks indicate significant differences (* *p* < 0.05; the Mann–Whitney U test)

To investigate the replication kinetics of rMDVs in vivo, we quantified the viral loads in the whole blood of chickens in the first and second experiments using qPCR. In both experiments, the viral load in the blood of chickens infected with rRB-1B_Meq tended to be higher than that of chickens infected with rRB-1B_Meq77/80 (Fig. 4A and B), even though the values were statistically significantly different at some time points and had high variability. In addition, the viral loads in the spleen, thymus, and feather tips of the chickens were also compared. The viral load in the spleens of rRB-1B_Meq-infected chickens tended to be higher than that in the rRB-1B_Meq77/80-infected chickens at 28 and 35 dpi (Fig. 4C). Interestingly, the thymus of rRB-1B_Meq77/80-infected chickens showed a high viral load at 7 dpi, which subsequently decreased and then became significantly lower than that of rRB-1B_Meq-infected chickens at 35 dpi (Fig. 4D). No significant differences in the viral load were detected in the feather tips of the rMDV-infected groups (Fig. 4E).

### Dynamics of CD4+ T cells and Meq^+^ cells in the peripheral blood mononuclear cells (PBMCs) and spleens of chickens infected with rMDVs

**Fig. 5:**
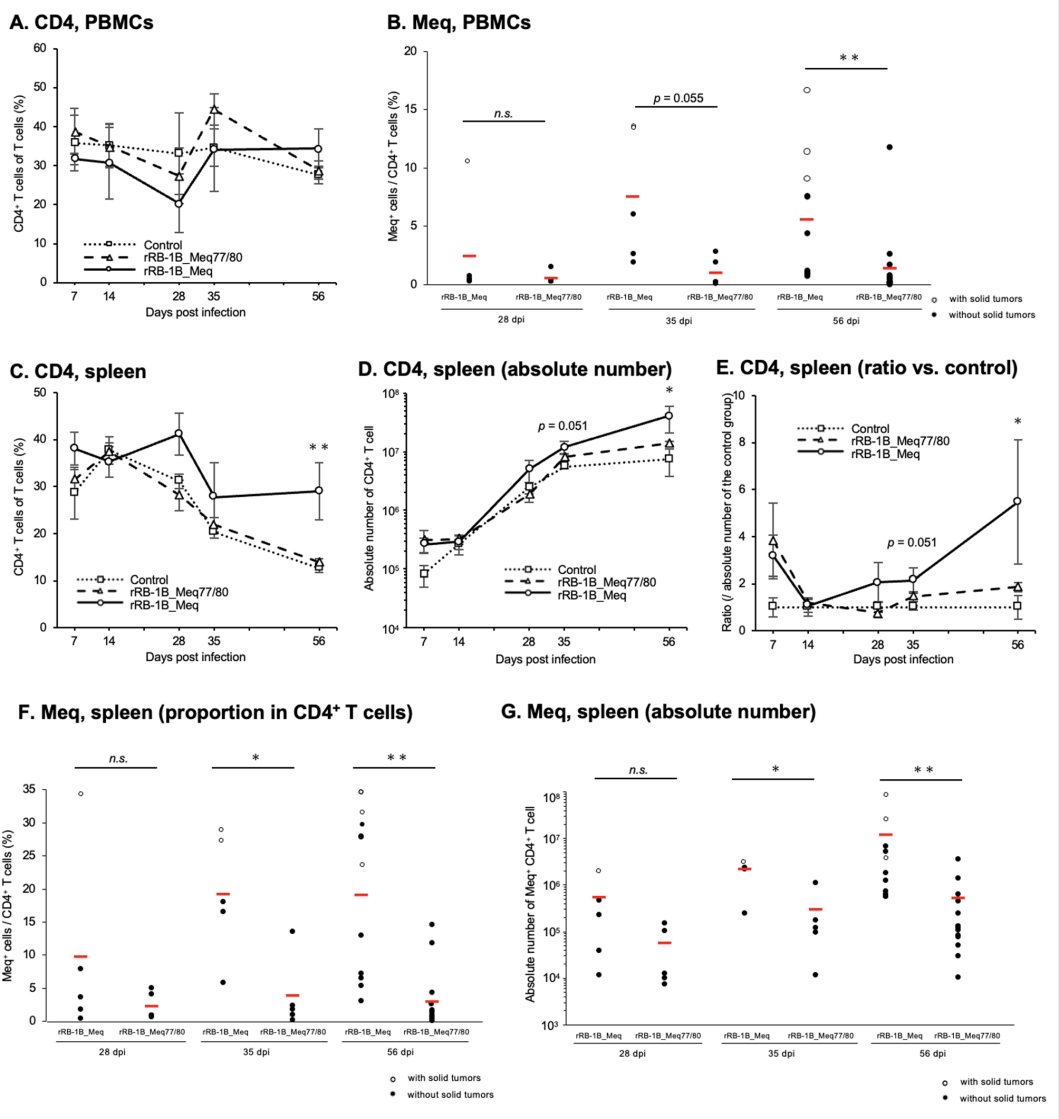
Dynamics of CD4^+^ T cells and Meq^+^ cells in chickens infected with rMDVs. Shown are the dynamics of CD4^+^ cells and Meq^+^ from the peripheral blood mononuclear cells (PBMCs) and spleens of chickens infected with rRB-1B-Meq or rRB-1B-Meq77/80 at each time point during the experimental period. The percentages of CD4^+^ T cells in the T cell population from (**A**) PBMCs and (**C**) spleens, and Meq^+^ cells in the CD4^+^ T cell population from (**B**) PBMCs and (**F**) spleens were analyzed. For the rRB-1B_Meq-infected group, the white and black dots denote chickens with and without solid tumors in visceral organs, respectively. Absolute numbers of (**D**) CD4^+^ T cells and (**G**) Meq^+^ CD4^+^ T cells in the spleen were analyzed. The ratios of absolute numbers of (**E**) CD4^+^ T cells in the spleen relative to the control group were analyzed. Asterisks indicate significant differences (* *p* < 0.05, **p < 0.01; Dunn’s test in A, C, D, and E. Mann–Whitney U test in **B**, **F**, and **G**). Significant differences were observed at 56 dpi (rRB-1B_Meq vs. control, *p* = 0.0015; rRB-1B_Meq77/80 vs. rRB-1B_Meq, *p* = 0.013) in (**C**), at 56 dpi (rRB-1B_Meq vs. control, *p* = 0.018) in (**D** and **E**)

Next, we investigated the dynamics of T cell subsets in chickens challenged with rMDVs by analyzing their PBMCs and spleens at each time point in the first experiment. MDV transforms CD4^+^ T cells into lymphoma cells, which highly express Meq [48]. In this experiment, no increase in the proportion of CD4^+^ T cells was observed in the PBMCs of chickens infected with either rMDV (Fig. 5A). The samples were collected from randomly selected chickens at each time point; in particular, the rRB-1B_Meq-infected group included chickens with or without tumors. Therefore, differences in the proportion of CD4^+^ T cells between the groups may not be observed due to high variability. However, in PBMCs from rRB-1B_Meq-infected chickens, the proportion of Meq⁺ cells among CD4⁺ T cells was significantly higher than that in rRB-1B_Meq77/80-infected chickens at 56 dpi, indicating an expansion of MDV-transformed cells (Fig. 5B). In contrast, although the proportion of Meq⁺ cells in PBMCs from rRB-1B_Meq77/80-infected chickens also tended to increase over time, the rate of increase was markedly slower compared to that in the rRB-1B_Meq group.

The spleens of chickens infected with rRB-1B_Meq showed a significantly higher proportion of CD4^+^ T cells than rRB-1B_Meq77/80-infected chickens at 56 dpi (rRB-1B_Meq77/80 vs. rRB-1B_Meq, *p* = 0.013) (Fig. 5C). Additionally, the absolute number of CD4^+^ T cells in the spleens of chickens in the rRB-1B_Meq-infected group was higher than that in the control group at 35 and 56 dpi; however, the absolute number of CD4^+^ T cells in the rRB-1B_Meq77/80-infected group tended to be lower than that in the rRB-1B_Meq-infected group (Fig. 5D and E). Furthermore, while no significant differences were observed in the proportion of Meq⁺ cells within the CD4⁺ T cell population in the spleens of the two infected groups at 28 dpi, the proportion of Meq⁺ cells in the rRB-1B_Meq77/80-infected group was significantly lower than that in the rRB-1B_Meq-infected group at 35 and 56 dpi (Fig. 5F). Notably, however, relatively high proportions of Meq⁺ cells were detected in one chicken at 35 dpi and two chickens at 56 dpi in the rRB-1B_Meq77/80-infected group. In addition, two chickens that exhibited open-mouth breathing at 44 dpi also showed a relatively high proportion of Meq⁺ cells within CD4^+^ T cells (Table 4). Similarly, although the absolute number of Meq⁺ CD4⁺ T cells tended to increase in both groups, the absolute number of Meq⁺ CD4⁺ T cells in the rRB-1B_Meq77/80-infected group was significantly lower than that in the rRB-1B_Meq-infected group at 35 and 56 dpi (Fig. 5G).

**Table 4:** Results of flow cytometric analysis of the spleen of rRB-1B_Meq77/80-infected chickens exhibiting open-mouth breathing in the first experimental infection

	CD4^+^/T cell ratio	Meq^+^/CD4^+^T cell ratio
rRB-1B_Meq77/80 #1 (44 dpi)	12.2%	4.42%
rRB-1B_Meq77/80 #2 (44 dpi)	12.8%	8.07%
rRB-1B_Meq77/80 (mean, 56 dpi, *n* = 14)	21.9%	3.02%
rRB-1B_Meq with tumor (mean; 28, 35, and 56 dpi; *n* = 6)	56.4%	30.0%

### Dynamics of CD8^+^ T cells and γδ T cells in the PBMCs and spleens of chickens infected with rMDVs

**Fig. 6:**
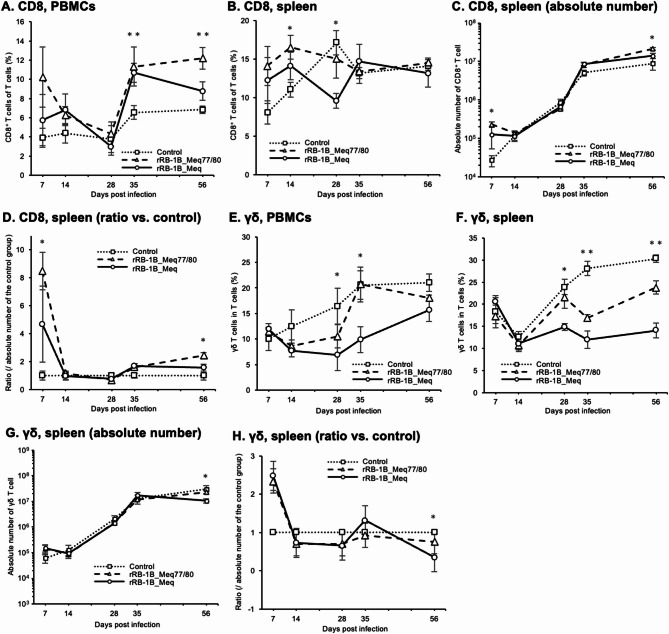
Dynamics of CD8^+^ T cells and γδ T cells in chickens infected with rMDVs. Shown are the dynamics of CD8^+^ cells and γδ T cells from the peripheral blood mononuclear cells (PBMCs) and spleens of chickens infected with rRB-1B-Meq or rRB-1B-Meq77/80 at each time point during the experimental period. The percentages of CD8^+^ T cells in the T cell population from (**A**) PBMCs and (**B**) spleens and γδTCR^+^ T cells in the T cell population from (**E**) PBMCs and (**F**) spleens were analyzed. Absolute numbers of (**C**) CD8^+^ T cells and (**G**) γδTCR^+^ T cells in the spleen were analyzed. The ratios of absolute numbers of (**D**) CD8^+^ T cells and (**H**) γδTCR^+^ T cells in the spleen relative to the control group were analyzed. Asterisks indicate significant differences (* *p* < 0.05, **p < 0.01; Dunn’s test). Significant differences were observed at 35 dpi (rRB-1B_Meq77/80 vs. control, *p* = 0.048; rRB-1B_Meq vs. control, *p* = 0.016) and 56 dpi (rRB-1B_Meq77/80 vs. control, *p* = 0.0013; rRB-1B_Meq77/80 vs. rRB-1B_Meq, *p* = 0.039) in (**A**), at 14 dpi (rRB-1B_Meq77/80 vs. control, *p* = 0.043) and 28 dpi (rRB-1B_Meq vs. control, *p* = 0.011) in (**B**), at 7 dpi (rRB-1B_Meq77/80 vs. control, *p* = 0.0070) and 56 dpi (rRB-1B_Meq77/80 vs. control, *p* = 0.0091; rRB-1B_Meq77/80 vs. rRB-1B_Meq, *p* = 0.047) in (**C and D**), at 28 dpi (rRB-1B_Meq vs. control, *p* = 0.043), 35 dpi (rRB-1B_Meq vs. control, *p* = 0.036; rRB-1B_Meq77/80 vs. rRB-1B_Meq, *p* = 0.043) in (**E**), at 28 dpi (rRB-1B_Meq vs. control, *p* = 0.024), 35 dpi (rRB-1B_Meq vs. control, *p* = 0.017; rRB-1B_Meq77/80 vs. rRB-1B_Meq, *p* = 0.060), at 56 dpi ((rRB-1B_Meq vs. control, *p* = 0.0004; rRB-1B_Meq77/80 vs. rRB-1B_Meq, *p* = 0.0016) in (**F**), and at 56dpi (rRB-1B_Meq vs. control, *p* = 0.035; rRB-1BMeq77/80 vs. rRB-1B_Meq, *p* = 0.0049) in (**G and H**)

We also analyzed the dynamics of CD8^+^ T cells, which play pivotal roles in cellular immunity against MDV infection and tumor cells [49]. The proportion of CD8^+^ T cells tended to be higher at 7 and 14 dpi in PBMCs from chickens infected with both rMDVs. This difference was not statistically significant; however, a significant increase was observed at 35 and 56 dpi compared to that in the control group (Fig. 6A). Notably, the proportion of CD8^+^ T cells in the rRB-1B_Meq77/80-infected group was significantly higher than that in the rRB-1B_Meq-infected groups at 56 dpi (rRB-1B_Meq77/80 vs. rRB-1B_Meq, *p* = 0.039). Moreover, the CD8^+^ T cell percentage in the spleens of rRB-1B_Meq77/80-infected chickens was significantly elevated at 14 dpi compared to that in the control group (Fig. 6B). Conversely, the percentage of CD8^+^ T cells in the rRB-1B_Meq-infected group was significantly lower than that in the control group at 28 dpi. Additionally, the absolute number of CD8^+^ T cells in the spleens of chickens in the rRB-1B_Meq77/80 group was significantly higher than that in the control group at 7 and 56 dpi and higher than that in the rRB-1B_Meq-infected group at 56 dpi (rRB-1B_Meq77/80 vs. rRB-1B_Meq, *p* = 0.047) (Fig. 6C and D).

Next, we examined the proportion of γδ T cells, which are known to have protective functions against MD in chickens vaccinated with the CVI988 strain [50, 51]. The percentage of γδ T cells in PBMCs from both rMDV-infected groups tended to be lower than that of the control group at 14 and 28 dpi (Fig. 6E). However, the proportion of γδ T cells in the rRB-1B_Meq-infected group was significantly lower than that of the rRB-1B_Meq77/80-infected group at 35 dpi (rRB-1B_Meq77/80 vs. rRB-1B_Meq, *p* = 0.043) in addition to the control group. Similarly, the proportion of γδ T cells in the spleens of chickens in the rRB-1B_Meq-infected group was significantly lower than that in the control group from 28 to 56 dpi. The γδ T cell proportion in the spleens of the rRB-1B_Meq77/80-infected group also tended to be lower than that of the control group at 35 and 56 dpi; however, it was only significantly higher than that in the rRB-1B_Meq-infected group at 56 dpi (rRB-1B_Meq77/80 vs. rRB-1B_Meq, *p* = 0.0016) (Fig. 6F). Furthermore, the absolute number of γδ T cells in the spleens of the rRB-1B_Meq-infected group was significantly lower than that in the rRB-1B_Meq77/80 at 56 dpi (rRB-1B_Meq77/80 vs. rRB-1B_Meq, *p* = 0.0049) (Fig. 6G and H). Collectively, these results suggest that no increase in the number of CD4^+^ T cells was observed in the spleen of the rRB-1B_Meq77/80-infected group, whereas the rRB-1B_Meq77/80 group showed a higher number of CD8^+^ T cells in both PBMCs and spleens. Furthermore, although the γδ T cell numbers were lower in both rMDV-infected groups compared to the control group, the RB-1B_Meq77/80-infected group showed a higher proportion of γδ T cells than the RB-1B_Meq-infected group in accordance with disease progression.

### Dynamics of T cell subsets in the thymus and bursa of fabricius of chickens infected with rMDVs

**Fig. 7:**
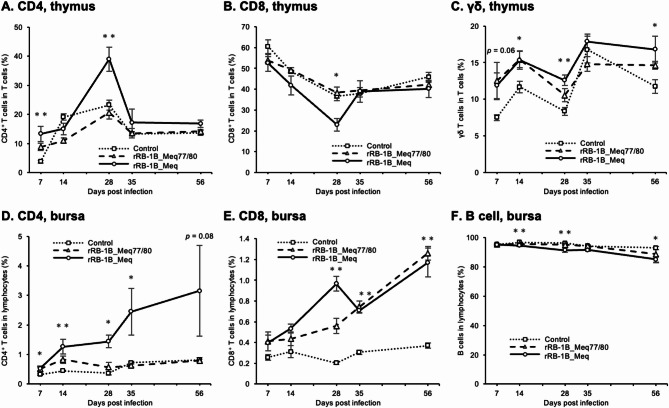
Dynamics of T cell subsets in the thymus and bursae of chickens infected with rMDVs. The dynamics of CD4^+^ cells, CD8^+^ cells, and γδ T cells from the thymus and bursae of chickens infected with rRB-1B-Meq or rRB-1B-Meq77/80 at each time point. The percentages of (**A**) CD4^+^ T cells, (**B**) CD8^+^ T cells, and (**C**) γδTCR^+^ T cells in the T cell population from the thymus were analyzed. The percentages of (**D**) CD4^+^ T cells, (**E**) CD8^+^ T cells, and (F) γδTCR^+^ T cells in the lymphocyte population from the bursa of Fabricius were also analyzed. Asterisks indicate significant differences (** *p* < 0.01, * *p* < 0.05; Dunn’s test). Significant differences were observed at 7 dpi (rRB-1B_Meq vs. control, *p* = 0.0028; rRB-1B_Meq77/80 vs. control, *p* = 0.042), and 28 dpi (rRB-1B_Meq vs. control, *p* = 0.0031; rRB-1B_Meq77/80 vs. rRB-1B_Meq, *p* = 0.035) in (**A**), at 28 dpi (rRB-1B_Meq vs. control, *p* = 0.011; rRB-1B_Meq77/80 vs. rRB-1B_Meq, *p* = 0.043) in (**B**), at 14 dpi (rRB-1B_Meq77/80 vs. control, *p* = 0.036), 28 dpi (rRB-1B_Meq77/80 vs. control, *p* = 0.0031), and 56 dpi (rRB-1B_Meq vs. control, *p* = 0.0053) in (**C**), at 7 dpi (rRB-1B_Meq vs. control, *p* = 0.024; rRB-1B_Meq77/80 vs. control, *p* = 0.046), 14 dpi (rRB-1B_Meq vs. control, *p* = 0.0028; rRB-1B_Meq77/80 vs. control, *p* = 0.042), 28 dpi (rRB-1B_Meq vs. control, *p* = 0.0044), and 35 dpi (rRB-1B_Meq vs. rRB-1B_Meq77/80, *p* = 0.018) in (**D**), at 28 dpi (rRB-1B_Meq vs. control, *p* = 0.007), 35 dpi (rRB-1B_Meq vs. control, *p* = 0.016; rRB-1B_Meq77/80 vs. control, *p* = 0.0086), and 56 dpi (rRB-1B_Meq vs. control, *p* = 0.0075; rRB-1B_Meq77/80 vs. control, *p* = 0.0020) in (**E**), and at 14 dpi (rRB-1B_Meq vs. control, *p* = 0.0013), 28 dpi (rRB-1B_Meq vs. control, *p* = 0.0017), and 56 dpi (rRB-1B_Meq vs. control, *p* = 0.021; rRB-1B_Meq77/80 vs. control, *p* = 0.026) in (**F**)

As rMDVs caused atrophy in both the thymus and bursa of rMDV-infected chickens (Fig. 3A and B), we investigated whether rMDV infection affected the proportion of lymphocytes in these lymphoid organs. The proportion of CD4^+^ T cells (CD3^+^CD4^+^CD8^−^) within the T cell population in the thymus of both rMDV-infected chickens was significantly higher than that of the control group at 7 dpi. The proportion of CD4^+^ T cells in the thymus of chickens infected with rRB-1B_Meq77/80 was significantly lower than that in the rRB-1B_Meq-infected group at 28 dpi (rRB-1B_Meq77/80 vs. rRB-1B_Meq, *p* = 0.035), whereas no difference between the control group and the rRB-1B_Meq77/80-infected group was observed from 14 dpi (Fig. 7A). The proportion of CD4^+^ T cells within the T cell population in the thymus of chickens infected with rRB-1B_Meq was considerably higher than those in the other two groups from 28 to 56 dpi. In contrast, the proportion of CD8^+^ T cells (CD3^+^CD4^−^CD8^+^) in the thymus of chickens in the rRB-1B_Meq-infected group decreased at 28 dpi compared to those in the rRB-1B_Meq77/80-infected group (rRB-1B_Meq77/80 vs. rRB-1B_Meq, *p* = 0.043). No difference between the control and rRB-1B_Meq77/80-infected groups was observed throughout the experimental period (Fig. 7B). In contrast to the results observed in PBMCs and the spleen, the proportion of γδ T cells in the thymus of both rMDV-infected groups tended to be higher throughout the experimental period compared to the control group (Fig. 7C). However, the RB-1B_Meq77/80-infected group tended to exhibit a lower proportion of γδ T cells from 28 to 56 dpi, compared to that in the rRB-1B_Meq-infected group.

We also observed that the proportion of CD4^+^ T cells among the lymphocytes in the bursa of rRB-1B_Meq-infected chickens was significantly higher than that in the bursa of control chickens throughout the experimental period (Fig. 7D). In contrast, the proportion of CD4^+^ T cells in the bursa of rRB-1B_Meq77/80-infected chickens significantly increased at 7 and 14 dpi compared to that in the control group, but did not show a significant increase thereafter. Additionally, compared with the control group, the proportion of CD8^+^ T cells in rRB-1B_Meq-infected chickens significantly increased from 28 dpi; similarly, the proportion of CD8^+^ T cells in the rRB-1B_Meq77/80-infected group significantly increased from 35 dpi (Fig. 7E). Furthermore, the proportion of B cells among lymphocytes in rRB-1B_Meq-infected chickens was significantly reduced from 14 to 56 dpi compared to that in the control group, while a significant reduction was observed in rRB-1B_Meq77/80-infected chickens at 56 dpi compared to that in the control group (Fig. 7F).

### Histopathological analysis of chickens infected with rMDVs

**Fig. 8:**
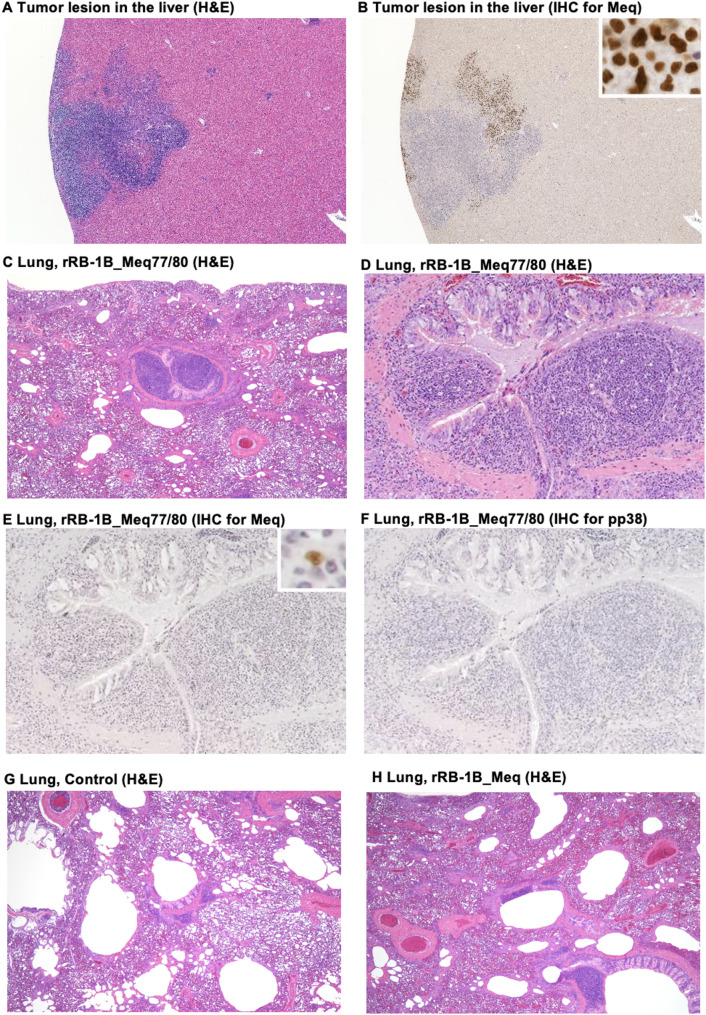
Histopathological analysis of chickens infected with rMDVs. (**A**) Hematoxylin and eosin (**H&E**) staining and (**B**) immunohistochemistry (IHC) staining of a tumor lesion from the liver of an rRB-1B_Meq77/80-infected chicken using anti-Meq monoclonal antibodies. The small panel indicates the presence of Meq-positive cells. (**C-D**) H&E, (**E**) IHC staining for Meq, and (**F**) IHC staining for pp38 in bronchus-associated lymphoid tissue (BALT) in the lung of an rRB-1B_Meq77/80-infected chicken. (**C**) shows a low-magnification image, and (**D**) shows a high-magnification image. Also shown are H&E-stained sections of a lung from a chicken in (**G**) the control group and (**D**) the rRB-1B_Meq-infected group

Next, we performed histopathological analysis of chickens at 56 dpi in the second animal experiment (control group: *n* = 2, rRB-1B_Meq77/80-infected group: *n* = 5, rRB-1B_Meq-infected group: *n* = 3). In the rRB-1B_Meq-infected group, three chickens exhibited solid tumor formation in the liver, kidneys, and gonads, and extensive infiltration of Meq-positive cells was observed in various organs (Table 5). In contrast, only two chickens infected with rRB-1B_Meq77/80 developed small solid tumors in the spleen, lungs, and liver, and the extent of lymphoma cell infiltration in each organ was markedly reduced compared to that in chickens infected with rRB-1B_Meq (Table 5). Additionally, the tumor lesions in the livers of chickens in the rRB-1B_Meq77/80-infected group consisted of Meq-positive cells and infiltrating lymphocytes (Fig. 8A and B), which were not observed in the rRB-1B_Meq-infected group.

In the rRB-1B_Meq77/80-infected group, four chickens exhibited hyperplasia in bronchus-associated lymphoid tissue (BALT) in the lung, characterized by marked infiltration and proliferation of lymphoid cells within the follicles and interfollicular regions (Fig. 8C and D). Although a small number of Meq-positive lymphocytes were detected at sites different from the tumor lesions in some organs, such as the spleens and thymus of two chickens, most of these lymphocytes were negative for Meq (Table 5; Fig. 8E), suggesting inflammation with occasional airway stenosis in the lungs. Immunohistochemistry (IHC) analysis showed no cells positive for pp38, a viral antigen expressed in infected cells during lytic replication, in the lungs of rRB-1B_Meq77/80 (Fig. 8F). In contrast, no BALT hyperplasia was detected in chickens in the control and the rRB-1B_Meq-infected groups (Fig. 8G and H). However, extensive infiltration of Meq-positive T cells was observed in the lungs of the chickens infected with rRB-1B_Meq (Table 5).

Furthermore, intranuclear inclusion bodies were detected in the skin of all chickens infected with rRB-1B_Meq, and IHC analysis for pp38 identified pp38-positive cells in most feather follicle epithelial cells (mean proportion of feathers with pp38-positive cells: neck skin, 47%; thigh skin, 73%) (Table 5). In contrast, no intranuclear inclusion bodies were observed in chickens infected with rRB-1B_Meq77/80, and the number of pp38-positive cells was significantly lower (neck skin, 14%; thigh skin, 0%). Thus, the results of our IHC analysis suggest that rRB-1B_Meq77/80 exhibits reduced tumorigenic potential and mild disease progression compared with rRB-1B_Meq. In contrast, chickens infected with rRB-1B_Meq77/80 seem to exhibit severe lung inflammation, whereas this finding was absent in chickens infected with rRB-1B_Meq.

**Table 5:** Summary of the results of the histopathological examination of tissues from chickens infected with Recombinant marek’s disease virus

Virus	Sample number	IHC	Spleen	Bursa of Fabricius	Thymus	Proventriculus	Lung	Brain	Cervical peripheral nerves	Cervical skin	Remarks
rRB-1B_ Meq	#1	Meq	++: Tumor lesions	+: Muscular layer, interfollicular region, follicles	++: Medulla +: Adipose tissue	++: Mucosa +: Muscular layer, serous membrane	+: Whole area	++: Perivascular region	++: Nerves	+++: Feather pulp ++: Skin stroma +: Adipose tissue, follicular epithelium	This chicken displayed leg paralysis. Tumor formation was observed in the testis. Enlargement of the sciatic nerve was noted. pp38 + follicular epithelium: 32% (6/19).
		pp38	+: Tumor lesions	+: Follicles	-	-	-	-	-	++: Follicular epithelium	
	#2	Meq	+++: Tumor lesions	+++: Interfollicular regions +: Muscle layer, follicles	+++: Medulla, perivascular lymphocytic clusters +: Adipose tissue	+++: Mucosa, serous membrane +: Muscle layer	+++: Tumor lesions	+++: Tumor lesions +: Meninges, perivascular regions	+: Nerves, adipose tissue	++: Follicular epithelium, skin stroma +: Feather pulp, adipose tissue	This chicken exhibited depression. Tumor formation was noted in the liver, kidneys, and ovaries. The bursa of Fabricius and the thymus were atrophied. pp38 + follicular epithelium: 61% (14/23).
		pp38	+: Tumor lesions	+: Follicles	+: Medulla	+: Serous membrane	+: Tumor lesions	-	-	++: Follicular epithelium	
	#3	Meq	+++: Tumor lesions	+++: Muscle layer, Interfollicular regions +: Follicles	++: Medulla, adipose tissue +: Cortex	+++: Serous membrane ++: Mucosa +: Muscle layer	+++: Tumor lesions ++: Whole area	+: Meninges, perivascular regions	+: Nerves, adipose tissue	++: Follicular epithelium, skin stroma +: Feather pulp, adipose tissue	Tumor formation was observed in the liver, kidneys, ovaries, and intestines. Ascites was present in the abdominal cavity. pp38 + follicular epithelium: 50% (12/24).
		pp38	++: Tumor lesions	+: Follicular medulla	+: Medulla	++: Serous membrane +: Mucosa , muscle layer	++: Tumor lesions +: Whole area	-	-	++: Follicular epithelium +: Feather pulp	
rRB-1B_ Meq77/80	#1	Meq	++: Tumor lesions	++: Tumor lesions	+: Medulla	+: Mucosa	+: Whole area, bronchial follicles	-	+: Nerves	+: Follicular epithelium, skin stroma	Tumor formation was observed in the liver and spleen. Lymphoid cell accumulation and lymphofollicular lesions were observed in the lungs. The body weight was lower than that of other chickens in the same group. pp38 + follicular epithelium: 21% (7/33).
		pp38	+: Tumor lesions	-	+: Medulla	-	+: Whole area (-: Bronchial follicles)	-	-	+: Follicular epithelium	
	#2	Meq	+: Parenchyma	+++: Tumor lesions +: Follicles	+: Medulla	+++: Mucosa, serous membrane ++: Muscle layer	+++: Tumor lesions	+: Meninges, perivascular regions	+++: Tumor lesions in adipose tissue +: Nerves	++: Feather pulp +: Skin stroma	Tumor formation was observed in the kidneys, proventriculus, and lungs. The body weight was lower than that of other chickens in the same group. pp38 + follicular epithelium: 0% (0/10).
		pp38	-	+: Tumor lesions	-	+: Tumor lesions	+: Tumor lesions	-	-	-	
	#3	Meq	+: Parenchyma	+: Follicular cortex, medulla	+: Medulla	+: Mucosa, muscle layer, Serous membrane	+: Whole area, bronchial follicles	-	+: Nerves	+: Follicular epithelium, feather pulp, skin stroma	Tumor formation was observed in the liver. Lymphoid cell accumulations and lymphofollicular lesions were observed in the lungs. pp38 + follicular epithelium: 21% (3/14).
		pp38	-	+: Follicles	-	+: Muscle layer	-	+: Parenchyma	-	+: Follicular epithelium	
	#4	Meq	-	-	+: Medulla	-	-	-	-	+: Skin stroma	Open-mouth breathing, lymphoid cell accumulation, and lymphofollicular lesions in the lungs were observed. pp38 + follicular epithelium: 0% (0/37).
		pp38	-	-	-	-	-	-	-	-	
	#5	Meq	-	-	-	-	-	-	-	-	Open-mouth breathing, lymphoid cell accumulation, and lymphofollicular lesions in the lungs were observed. pp38 + follicular epithelium: 0% (0/10).
		pp38	-	-	-	-	-	-	-	-	
The IHC results indicate the percentage of Meq/pp38-positive cells per five fields of view. +, < 30%; ++, 30–80%; +++, > 80%;IHC, immunohistochemistry											

### Effects of amino acid substitutions in the basic region of Meq on its transcriptional regulation activity

**Fig. 9:**
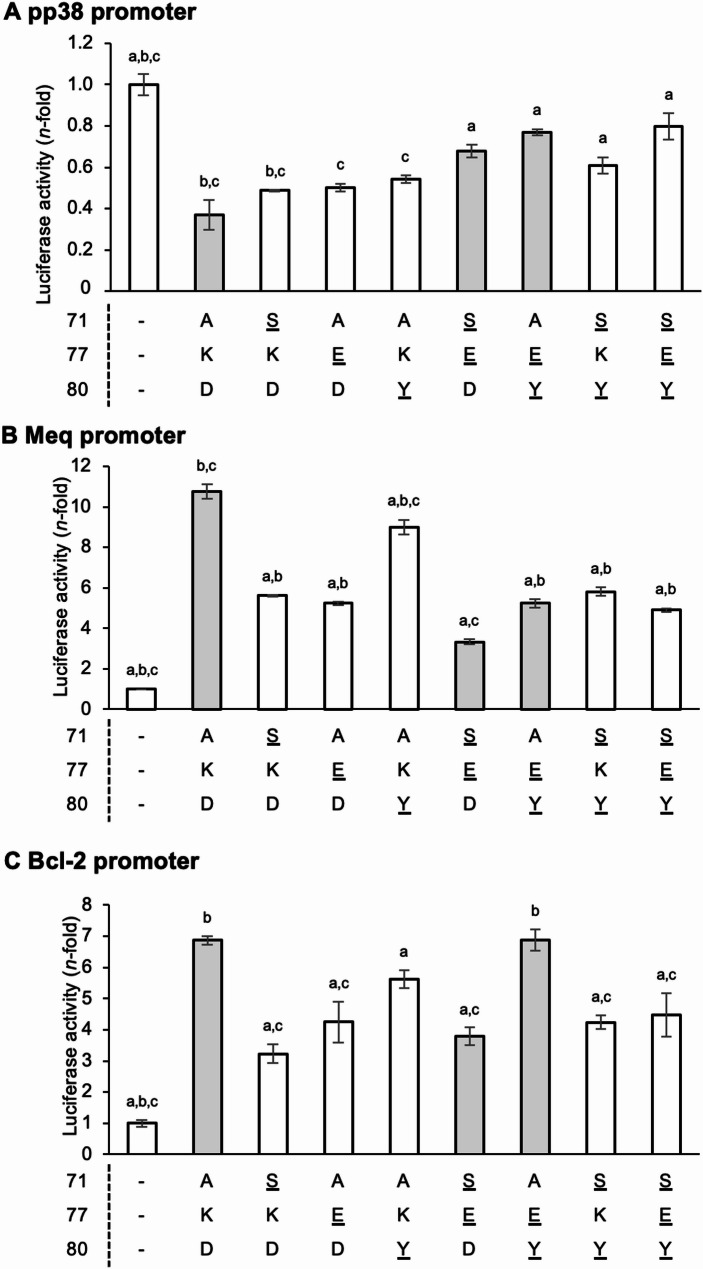
Analysis of the effect of polymorphisms in the basic region on Meq-induced transcriptional regulation. (**A**) Transrepression effects of the Meq isoforms. The transrepression effects of each Meq isoform on *pp38* promoter-driven luciferase activities were compared. DF-1 cells in each well were transfected with 300 ng of expression plasmids for each Meq isoform, 500 ng of reporter plasmid, and 5 ng of the control pRL-TK plasmid. Luciferase activities were analyzed 24 h post-transfection. Firefly luciferase activity is expressed relative to the mean basal activity in the presence of pCI-neo after normalization to *Renilla* luciferase activity. (**B, C**) Transactivation activities of the Meq isoforms. The transactivation activities of each Meq isoform on the (**B**) *meq* promoter- and (**C**) *bcl-2* promoter-driven luciferase activities were compared. DF-1 cells in each well were transfected with 300 ng of expression plasmids for each Meq isoform, 200 ng of c-Jun expression plasmid, 500 ng of reporter plasmid, and 5 ng of the control pRL-TK plasmid. The gray bars indicate Meq isoforms with the amino acid sequences in the basic region found in the field strains. The tables below each graph show the amino acid residues at positions 71, 77, and 80 of each Meq isoform. The underlined amino acid residues indicate differences from RB-1B-Meq (71 A, 77 K, 80D). The value of basal control in the left side reflects cells transfected with an empty pCI-neo vector, not with the c-Jun expression vector. Three independent experiments were performed in triplicate. Error bars indicate the standard deviations. (a: *p* < 0.01, vs. Meq (71 A, 77 K, 80D); b: *p* < 0.01, vs. Meq (71 S, 77E, 80D); c: *p* < 0.01, vs. Meq (71 A, 77E, 80Y); Tukey’s multiple comparison test)

We previously reported that the insertion of a 60-amino-acid sequence in the transactivation domain of Meq enhances the transactivation activity of various host and viral promoters [37]; therefore, we hypothesized that polymorphisms in Meq may also affect its transcriptional regulation activity. To investigate the mechanisms by which the amino acid substitutions at positions 77 and 80 reduce MDV virulence, we examined their effects on transcriptional regulation using reporter assays for the *pp38*, *meq*, and *bcl-2* promoters. In addition to polymorphisms at positions 77 and 80, an alanine-to-serine substitution at position 71 is commonly found in the vaccine strain CVI988 and recent field strains isolated in Brazil and Iraq [52, 53]. Therefore, we also included this third mutation and generated single, double, and triple mutant Meq expression plasmids based on RB-1B-Meq. Previous studies reported that Meq/Meq homodimers suppress *pp38* promoter activity [54]. To investigate the differences in the transrepressive effects of the different Meq mutants, we transfected a reporter plasmid containing the *pp38* promoter along with each Meq construct. All Meq isoforms exhibited transrepressive effects on the *pp38* promoter (Fig. 9A). Although the differences were not statistically significant, Meq isoforms with single mutations tended to exert decreased transrepression than the wild-type Meq. In contrast, all Meq isoforms with double and triple mutations showed significantly lower transrepression effects than wild-type Meq. These findings suggest that amino acid substitutions in the basic region of Meq observed in low-virulence strains showed a reduced transrepressive effect on the *pp38* promoter.

Given that the Meq/c-Jun heterodimer is reported to upregulate the expression of the *meq* and *bcl-2* promoters [25, 55, 56], we compared the transactivation activities of these Meq constructs with mutations in the basic regions of the *meq* and *bcl-2* promoters. Although all Meq isoforms enhanced the activity of the *meq* promoter, Meq isoforms with mutations showed significantly lower transactivation activities than wild-type Meq (Fig. 9B). Notably, the Meq isoform with mutations at positions 71 and 77, which had the same amino acid sequence as Meq in the CVI988 strain, exhibited the lowest transactivation activity among all the Meq isoforms investigated. In addition, all Meq isoforms increased the activity of the *bcl-2* promoter (Fig. 9C). However, the Meq isoforms with a single mutation at position 80 and a double mutation at positions 77 and 80 showed transactivation activity similar to that of wild-type Meq. Thus, these data suggest that certain combinations of amino acid polymorphisms in the basic region are associated with transcriptional regulation by Meq. However, the effects vary depending on the combination of mutations and the type of promoter.

## Discussion

In this study, we investigated the effects of amino acid polymorphisms in the basic region of *meq* on the virulence of MDV and the transcriptional regulation of Meq. The introduction of amino acid polymorphisms observed in low-virulence strains in the USA and Asian isolates into RB-1B-Meq reduced the transcriptional regulation functions of Meq. Further, rRB-1B_Meq77/80 exhibited a lower disease and tumor incidence than rRB-1B_Meq and caused different clinical manifestations, such as open-mouth breathing. Flow cytometric analysis revealed an increase in the proportion of CD8^+^ T cells in both PBMCs and the spleen of rRB-1B_Meq77/80-infected chickens. Histopathological analysis revealed lymphocytic hyperplastic bronchitis in rRB-1B_Meq77/80-infected chickens, suggesting that severe lung inflammation may underlie the respiratory signs observed during infection.

Although the replication ability between rMDVs was not significantly different in vitro, the rRB-1B_Meq-infected group exhibited higher viral loads in both the blood and spleen compared to the rRB-1B_Meq77/80-infected group. Similarly, an increase in Meq^+^ CD4^+^ T cells, which are believed to contain the MDV genome, was observed in rRB-1B_Meq-infected chickens by flow cytometric analyses, suggesting that the high viral load in the rRB-1B_Meq-infected group may reflect the proliferation of transformed CD4^+^ T cells. Conversely, even in the second experiment, in which the inoculum dose was increased from 5,000 to 10,000 pfu, the viral load in rRB-1B_Meq77/80-infected chickens did not increase, possibly because of the reduced transformation ability of Meq77/80 and lower proliferation of lymphoma cells, consistent with the observation that Meq^+^ CD4^+^ T cells did not increase in most rRB-1B_Meq77/80-infected chickens.

MDV can transform CD4^+^ T cells increasing their proliferation and the formation of lymphomas [48]. Therefore, the increased number of Meq^+^ CD4^+^ T cells in the rRB-1B_Meq-infected group at the late phase of infection implied the proliferation of MDV-transformed cells. In contrast, the proportion of Meq^+^ CD4^+^ T cells in both PBMCs and spleens of the rRB-1B_Meq77/80-infected group was significantly lower than that in the rRB-1B_Meq-infected group. Moreover, only a few Meq-positive cells were detected in the spleens of chickens infected with rRB-1B_Meq77/80 at different sites from the tumor lesions, compared to chickens infected with rRB-1B_Meq (Table 5). These findings suggest that rRB-1B_Meq77/80 may not efficiently establish a latent infection or drive replication of latent cells due to the reduced transformation ability of Meq77/80; subsequently, the infected cells may be eliminated by the immune system. However, in the rRB-1B_Meq77/80-infected group, one chicken at 35 dpi and two chickens at 56 dpi in the first experiment exhibited high proportions of Meq⁺ CD4^+^ cells. Similarly, Meq⁺ CD4^+^ cell proportions were elevated in the rRB-1B_Meq77/80-infected chickens that showed open-mouth breathing. Therefore, it is plausible that the rRB-1B_Meq77/80-infected chickens with high proportions of Meq⁺ CD4^+^ cells may have exhibited open-mouth breathing or solid tumor formation with more prolonged observation.

CD8^+^ T cells play a critical role in both antiviral and antitumor immunity against MDV infection [57, 58]. In rRB-1B_Meq77/80-infected chickens, both the number and proportion of CD8^+^ T cells increased during the acute and transformation phases, suggesting that these CD8^+^ T cells may respond to infected or transformed cells and contribute to the milder pathogenesis of rRB-1B_Meq77/80. However, the increase in the number of CD8^+^ T cells may be associated with inflammatory lesions in the lungs and cause different clinical manifestations, such as open-mouth breathing. Therefore, the potential association of the CD8^+^ T cells with the clinical manifestations of rRB-1B_Meq77/80 infection requires further investigation.

Tumor incidence was highly increased in the γδ T cell-knockout chickens in the experimental infection, highlighting the critical roles of γδ T cells in immunity against MDV infection [59]. Consistent with previous experimental infection using RB-1B [51], our data revealed that the absolute number of γδ T cells in the spleens of rRB-1B_Meq-infected chickens was reduced compared to that of the control group. The supernatant of splenocytes from MDV-infected chickens has also been shown to reduce the frequency of IFN-γ^+^ γδ T cells in vitro [60]. MDV-transformed cells can express prostaglandin E_2_ and programmed death-ligand 1, which can potentially create an immunosuppressive environment [61, 62]. Therefore, these immunosuppressive molecules can potentially reduce the number of γδ T cells. Further investigations are crucial for understanding the mechanisms of MDV pathogenesis, such as how immunosuppressive factors influence γδ T cells.

In this study, we found that amino acid substitutions in the basic region of low-virulence strains in the USA and Asian isolates reduced the transcriptional repression of the *pp38* promoter. Although a single substitution slightly decreased these transrepressive effects, double or triple substitutions led to a significant reduction in transcriptional repression, suggesting a potential synergistic effect of these mutations. Furthermore, Meq isoforms carrying glutamic acid and tyrosine at positions 77 and 80, respectively (indicated as gray bars in Fig. 9), reduced the transrepressive effects of the *pp38* promoter and the transactivation activity on the *meq* promoter compared to the wild-type RB-1B-Meq. Conversely, these substitutions did not affect the transactivation of the *bcl-2* promoter. These observations suggest that mutations at positions 77 and 80 may not uniformly reduce the binding affinity of Meq for all promoters, but may alter the specificity of Meq towards different promoters, thereby selectively influencing its regulatory functions. We previously demonstrated that polymorphisms in the Meq of highly virulent strains elicited enhanced transactivation activity, suggesting a correlation between Meq-mediated transcriptional regulation and MDV virulence [33, 37]. These mutations may affect the nuclear and nucleolar localization of Meq due to their proximity to these localization signals or the stoichiometry of its homo- to heterodimerization [54, 63, 64]. Moreover, as Meq interacts with multiple host and viral proteins via its basic region [27, 65], further investigation, such as conducting a proximity-dependent biotin labeling approach and co-immunoprecipitation, is required to determine the proteins interacting with Meq.

Furthermore, the Meq isoform with serine and glutamic acid at positions 71 and 77, respectively (shown as gray bars in Fig. 9), corresponding to the CVI988-Meq, exhibited lower transcriptional regulation function than the wild-type RB-1B-Meq. As the mutations at positions 77 and 80 reduced both the transactivation activity of Meq and the virulence of rRB-1B in this study, the amino acid residues at positions 71 and 77 can potentially contribute to the reduced transactivation activity and low virulence of the CVI988 strain. However, several highly virulent strains in Europe and Nigeria also contain glutamic acid and tyrosine at positions 77 and 80 (11), suggesting that the enhanced transactivation activity of Meq may not be necessary for increased virulence in all strains. In these strains, virulence may be influenced by compensatory mutations in other viral genes or alternative mechanisms independent of transactivation activity. For instance, Meq is known to interact with host proteins such as C-terminal binding protein and p53 through the basic region [27, 65]; therefore, polymorphisms in this region may affect the function of Meq via its interaction with these proteins. To determine whether mutations at positions 71 and 77 in Meq reduced the virulence of MDV, experimental infections using rMDV harboring Meq (71 S, 77E, 80D) are necessary.

Histopathological analysis revealed BALT hyperplasia in the lungs of rRB-1B_Meq77/80-infected chickens, which was not observed in either the control group or the rRB-1B_Meq-infected group. Therefore, the incidence of MD may be higher if histopathological examination was conducted on all infected chickens, which was difficult to detect from clinical signs alone. Moreover, two chickens in the rRB-1B_Meq77/80-infected group that showed open-mouth breathing at 44 dpi exhibited elevated Meq⁺ CD4^+^ cell proportions, suggesting an association between the elevation of Meq⁺ CD4^+^ cells and open-mouth breathing. The lymphocytic hyperplastic bronchitis in the lungs of the chickens observed in this study resembled that seen in *Mycoplasma* spp. infection. However, immunohistochemical staining for *Mycoplasma* was negative in all samples (data not shown). In contrast, there was minimal infiltration of Meq-positive lymphocytes into the proventriculus, gizzard, brain, or vagus nerve, suggesting that open-mouth breathing is not associated with digestive or nervous system disorders and that lung inflammation is the cause of this unique clinical sign. However, we could not clarify the mechanism by which rRB-1B_Meq77/80 causes lung inflammation. We considered two hypotheses to explain the pathogenesis of rRB-1B_Meq77/80. The first hypothesis is that a small number of transformed cells drives lung inflammation. Given the low transactivation activity of Meq77/80 and the presence of a few Meq^+^ lymphocytes within the lymphofollicular lesions in the lungs, a limited number of transformed cells may fail to create an immunosuppressive environment, resulting in lung inflammation. This is consistent with the observation that inflammatory cells were present in close proximity to Meq^+^ lymphocytes in the tumor lesion in the livers of rRB-1B_Meq77/80-infected chickens. The second hypothesis is that polymorphisms in the basic region alter the transcriptional targets of Meq. Differences in the amino acid sequences of the basic region involved in binding to genomic DNA may alter the affinity of Meq for its target regions and modify the transcriptomic profile, potentially enhancing inflammatory cytokine expression. To explore this hypothesis, further analysis is required to analyze the differences in target genes among the Meq isoforms using chromatin immunoprecipitation-sequencing and gel shift assays. Furthermore, direct comparison of the transcriptomes in infected cells using approaches such as single-cell RNA sequencing or the development of reporter viruses to enrich infected cells may provide valuable insights into the mechanisms underlying the pathological differences.

In the second experimental infection, two chickens in the rRB-1B_Meq77/80-infected group were found to have developed solid tumors at necropsy. Compared with the other rRB-1B_Meq77/80-infected chickens lacking tumors, these two birds were characterized by relatively higher viral loads and absolute numbers of Meq⁺ CD4⁺ T cells at 56 dpi (data not shown). IHC analysis for Meq revealed that the tumor lesions in the livers of rRB-1B_Meq77/80-infected chickens contained Meq-negative lymphocytes. Considering the increased number of CD8⁺ T cells in the rRB-1B_Meq77/80-infected group, effector T cells may infiltrate the tumor lesion. A previous study showed that differences in the amino acid sequence of Meq can affect cellular composition in the tumor tissue [66], potentially altering the transcriptome of transformed cells, including cytokine and chemokine expression. Thus, variations in the Meq sequence may modify the tumor microenvironment. Analyses using antibodies against macrophages, NK cells, or other lymphoid populations to assess substantial differences in tumor composition may reflect variations in the Meq coding sequence.

Furthermore, histopathological analysis of the skins of rRB-1B_Meq77/80-infected chickens revealed an absence of nuclear inclusion bodies and a significantly lower number of pp38-positive cells compared with those in rRB-1B_Meq-infected chickens. These findings indicate that disease progression in the rRB-1B_Meq77/80 birds may have been slower, potentially resulting in fewer infected T cells reaching the skin, thereby leading to diminished viral replication within the feather follicle epithelium and reduced horizontal transmission to naïve chickens. To assess the influence of amino acid polymorphisms at positions 77 and 80 on the efficiency of viral dissemination, it will be necessary to examine the transmission of the virus from infected to uninfected chickens.

Susceptibility to MD is affected by the genetic background and the presence of maternal antibodies. Commercial chickens possess maternal antibodies against MDV, which may delay the onset of MD; therefore, MD-susceptible SPF inbred chicken lines, such as the lines P or 7, are preferable for evaluating MDV virulence. In the present study, we conducted experimental infections using commercial chickens due to the limited availability of these SPF inbred lines in Japan. Indeed, compared to a study using SPF chickens [67], the peak of MD onset in our experiments was delayed by approximately one week. Nevertheless, thymic and bursal atrophy was observed in both rMDV-infected groups in our experiments, indicating that the effect of early cytolytic infection was not entirely masked due to the presence of maternal antibodies. However, it is important to note that the early phase of cytolytic infection may have been underestimated in this experimental setting.

In this study, we monitored the infected chickens for 8 weeks post-infection. It is thus plausible that if we had extended the period of monitoring, we may have observed increases in the incidence of tumors and mortality in the rRB-1B_Meq77/80-infected chickens. However, given the low virulence of rRB-1B_Meq77/80, it is also conceivable that rRB-1B_Meq77/80 would not induce further clinical signs in chickens. Consequently, further experimental infections, including observations over prolonged periods, are warranted to clarify these possibilities.

Although no significant difference in the survival rate was observed in the first experiment (*p* = 0.06, log-rank test), it was evident in the second (*p* < 0.01). The higher dose of rRB-1B_Meq used in the second experiment may have contributed to the increased mortality and tumor incidence observed, especially given the high oncogenicity of this strain. While previous studies using less virulent strains showed no dose-dependent effects [68, 69], our results suggest that the viral dose can influence disease outcomes in infections with highly oncogenic strains. Other factors, such as sample size, the presence of maternal antibodies, and genetic heterogeneity, may also have influenced the disease outcomes. However, in our previous experiments using rRB-1B_Meq, we consistently observed approximately 50% MD incidence and tumor formation [37, 44]. This consistency confirms that the observed variation is within acceptable experimental variability and does not compromise reproducibility.

## Conclusion

We demonstrated that mutations in the basic region of Meq observed in low-virulence strains in the USA and Asia reduced the transcriptional regulatory function of Meq and MDV virulence. In contrast, rRB-1B_Meq77/80 induced BALT hyperplasia in the lungs and seemed to induce different clinical manifestations, such as open-mouth breathing. This suggests that polymorphisms observed in the basic region of highly virulent strains in the USA, such as the RB-1B strain, may have contributed to the evolution toward higher virulence. In contrast, MDV isolates from Asian countries may have evolved differently from the MDV strains in the USA, potentially altering the clinical manifestations upon infection. Moreover, recent Asian isolates have been reported to possess mutations in PRRs and deletion sequences in the transactivation domain [7, 30, 31, 34, 70–72]. Therefore, to fully understand the relationship between the amino acid sequences of Meq and MDV virulence, it is essential to investigate the potential effects of a combination of mutations in other Meq domains. Notably, even just two amino acid substitutions in the basic region unexpectedly altered the clinical manifestations, such as open-mouth breathing. Therefore, field strains encoding Meq with the same amino acid residues at positions 77 and 80 as Meq77/80 may potentially cause similar clinical manifestations. In contrast to typical clinical manifestations such as lymphomas and neurological disorders, cases with respiratory signs caused by field strains with these polymorphisms may be overlooked, and the impact of MD on the poultry industry may be underestimated. Therefore, strengthening MDV surveillance in the field is crucial.

## Supplementary Information

Below is the link to the electronic supplementary material.

**Supplementary Material 1**: **Supplementary Fig. 1**. Gating strategy for the analysis of the dynamics of major T cell subsets in chickens infected with rMDVs. A representative gating. Strategy is shown to analyze the dynamics of γδ T cells, CD8^+^ T cells, CD4^+^ T cells, and meq+^+^ cells. Dead cells were excluded using the fixable viability dye eFluor780 (Thermo Fisher Scientific)

## Data Availability

No datasets were generated or analysed during the current study.

## Abbreviations

MDV: Marek’s disease virus
MD: Marek’s disease
rMDV: Recombinant Marek’s disease virus
NK: Natural killer
PRRs: Proline-rich repeats
bZIP: Basic leucine zipper
CEFs: Chicken embryo fibroblasts
BAC: Bacterial artificial chromosome
IRL: Internal repeat long
TRL: Terminal repeat long
RFLP: Restriction fragment length polymorphism
qPCR: Quantitative PCR
PBS: Phosphate-buffered saline
*icp4*: *Infected cell protein 4*
*i-nos*: *Inducible nitric oxide synthase*
mAb: Monoclonal antibody
FITC: Fluorescein-5-isothiocyanate
APC: Allophycocyanin
dpi: Days post-infection
pfu: Plaque**-**forming units
PBMCs: Peripheral blood mononuclear cells
Cy: Cyanine
vvMDV: Very virulent Marek’s disease virus
vv + MDV: Very virulent plus Marek’s disease virus
