# Supplementary figures and images for: Two distinct polymorphisms in the basic region of Meq protein of marek’s disease virus alter pathological progression and clinical manifestations
Source: Virol J. 2025 Sep 26;22:303. doi: 10.1186/s12985-025-02930-4 (PMC12466078; doi:10.1186/s12985-025-02930-4)

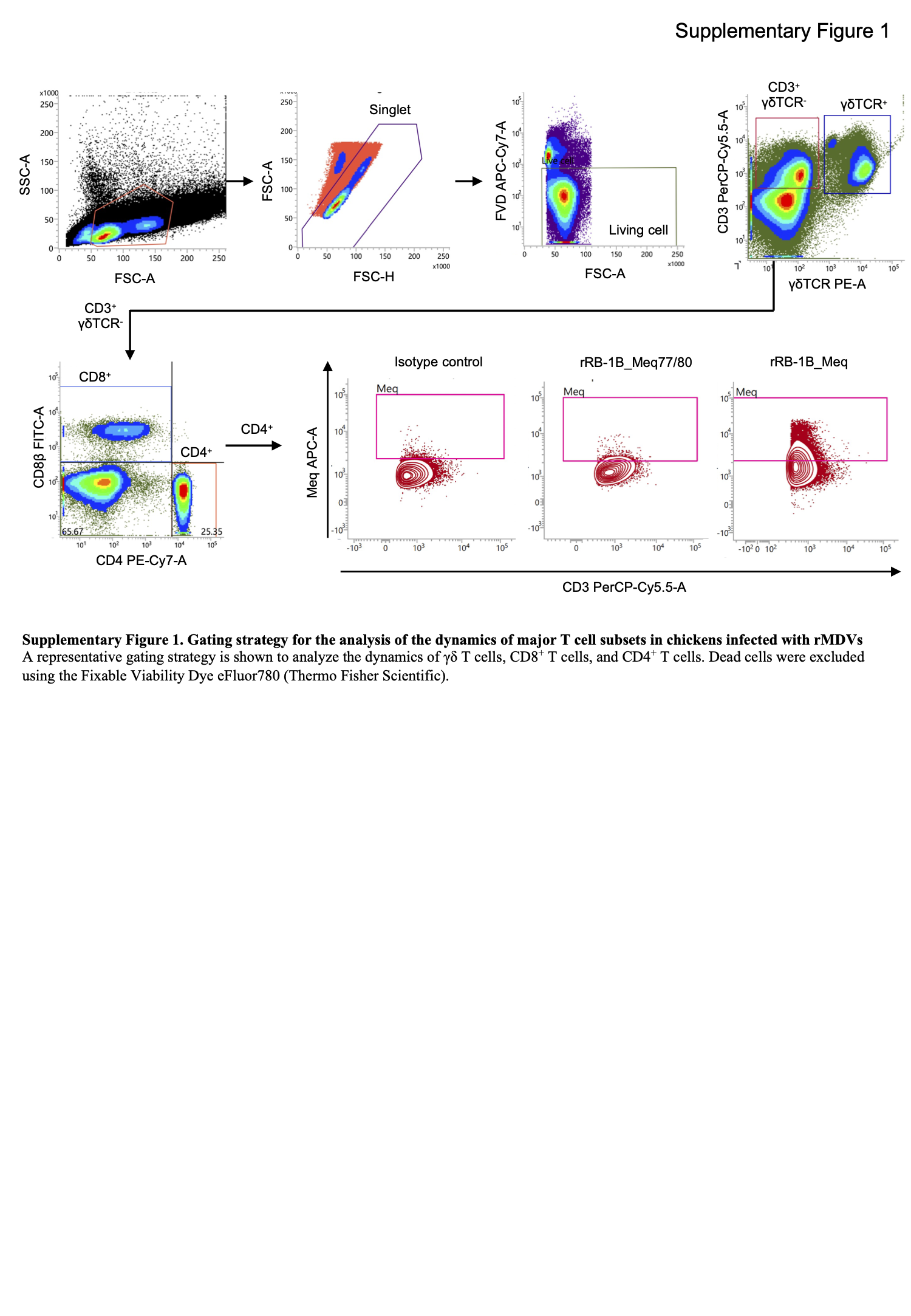

Supplement: Supplementary file 1 — Supplementary Material 1: Supplementary Fig. 1. Gating strategy for the analysis of the dynamics of major T cell subsets in chickens infected with rMDVs. A representative gating. Strategy is shown to analyze the dynamics of γδ T cells, CD8+ T cells, CD4+ T cells, and meq++ cells. Dead cells were excluded using the fixable viability dye eFluor780 (Thermo Fisher Scientific) [file 12985_2025_2930_MOESM1_ESM.tiff]
